# Small Noncoding RNAs Contribute to Sperm Oxidative Stress-Induced Programming of Behavioral and Metabolic Phenotypes in Offspring

**DOI:** 10.1155/2022/6877283

**Published:** 2022-06-06

**Authors:** Li Ren, Yining Xin, Xiaoxiao Sun, Yanwen Zhang, Yingqi Chen, Suyuan Liu, Bin He

**Affiliations:** ^1^Key Laboratory of Animal Physiology & Biochemistry, Ministry of Agriculture and Rural Affairs, College of Veterinary Medicine, Nanjing Agricultural University, Nanjing 210095, China; ^2^MOE Joint International Research Laboratory of Animal Health & Food Safety, Nanjing Agricultural University, Nanjing 210095, China

## Abstract

There is growing evidence that paternal environmental information alters small noncoding RNAs (sncRNAs) in sperm and in turn can induce alterations of metabolic and behavioral phenotypes of the next generation. However, the potential mediators of the effects remain to be elucidated. A great diversity of environmental insults and stresses can convergently induce the elevation of reactive oxygen species (ROS) in sperm; nonetheless, it remains unclear whether ROS mediates the biogenesis of sncRNAs in sperm and participates in the reprogramming of offspring phenotypes. Here, we show that ROS could induce the alteration of sncRNA profiles in sperm, especially for transfer RNA-derived small RNAs (tsRNAs) and ribosomal RNA-derived small RNAs (rsRNAs). Zygotic injection of 29-34 nt RNA fractions (predominantly tsRNAs and rsRNAs) from oxidative stress (OS) sperm could induce depressive-like and anxiety-like behaviors in male offspring. Moreover, zygotic injection with synthetic RNAs partially resembled OS sperm-induced depressive-like and anxiety-like behaviors in offspring. Male offspring maintained on a chow diet was found to develop impaired glucose tolerance and hyperactive hepatic gluconeogenesis, accompanied by the upregulation of hepatic gluconeogenic and lipolytic genes. Together, our results have shown that ROS-induced alteration of sncRNA profiles in sperm contributes to the alterations of behavioral and metabolic phenotypes of the offspring.

## 1. Introduction

Environmental conditions in which one generation experienced can manifest in the phenotypes of future generations, and the understanding of this mechanism has tremendous implications in not only basic biology, but also public health [1–9]. Interestingly, most of the paternal environmental stress, including mental stress, unhealthy diet, and toxin exposure can convergently induce the alteration of metabolic and behavioral phenotypes of the next generation [10–14]. This leads to the speculation whether a certain factor in sperm is mediated by these environmental stimuli. In fact, a wide range of stressful environmental stimuli including unhealthy diets (e.g., low protein and high fat), cigarette smoking, alcohol intake, heat stress, and toxicants (e.g., endocrine disruptors) increased reactive oxygen species (ROS) levels in sperm [15–19]. Physiological production of ROS in sperm can regulate essential functions such as capacitation, acrosome reaction, motility, hyperactivation, and sperm-oocyte fusion [20]. Meanwhile, ROS-induced sperm oxidative stress (OS) is known to induce male infertility [20–23]. Recently, Lane et al. have found that the elevation of sperm ROS concentration induces metabolic syndrome and obesity in the offspring [24]. It implies that ROS may serve as one of the mechanisms by which paternal information induced programming of phenotypes in offspring. However, a clear mechanism of this intergenerational effect remains to be elucidated.

Recently, sperm-borne small noncoding RNAs (sncRNAs) are being shown to transfer paternal epigenetic traits transgenerationally other than DNA [5, 6, 12–14, 25–31]. We and others have reported that 30-40 nucleotide (nt) RNA fractions in sperm, such as transfer RNA-derived small RNAs (tsRNAs) and ribosomal RNA-derived small RNAs (rsRNAs), as well as their modifications, could be rapidly altered by environmental inputs such as unhealthy diets and toxicants, which then contribute to the intergenerational inheritance of metabolic disorders [5, 6, 25, 29, 32, 33]. Also, sperm sncRNAs play a crucial role in the transgenerational modification of offspring addictive-like and anxiety-like behaviors [25, 31, 33]. ROS is known to be participate in epigenetic modifications including DNA methylation, histone modifications, and noncoding RNAs [34–37]. Recently, research has indicated that ROS induces cleavage of tRNAs into tsRNAs and the cleavage of rRNAs into rsRNAs by regulating their biogenesis pathways [38]. Paternal low-protein diet can elevate the production of ROS in male germ cells and induced expression changes in the liver of offspring and the biogenesis of sncRNA in sperm [39]. It can be of great significance to understand whether ROS is involved in the mediation of sncRNA biogenesis in mature sperm or participates in the reprogramming of phenotypes of the offspring.

In order to explore whether ROS-induced alteration of sncRNA profiles in sperm could contribute to reprogramming of phenotypes in offspring, we conducted a study which is aimed at investigating the effect of ROS on sncRNA profiles in mature sperm. Moreover, through the of a zygotic injection model, we provided direct evidence on the function of these sncRNAs in the alterations of metabolic and behavioral phenotypes of the offspring.

## 2. Materials and Methods

### 2.1. Sperm Treatment and Collection

Sperm which was from the vas deferens and caudal epididymis of mice was collected as described previously [24] and allowed to swim out in 1 mL of Whitten**'**s-HEPES-buffered medium at 37°C for 15 min [40]. Then, sperm from each mice were split into either control Whitten**'**s-HEPES-buffered medium or Whitten**'**s-HEPES-buffered medium supplemented with 1500 *μ*M H_2_O_2_ (this concentration increases ROS levels in sperm but does not affect sperm viability or the ability to bind and fertilize an oocyte [24]) for 3 h. After that, sperm were assessed for intracellular ROS levels, motility, and for RNA extraction.

### 2.2. Assessment of Mitochondrial ROS

To measure sperm ROS levels, sperm were incubated with 2 *μ*M MitoSox Red (MSR, Molecular Probes, Eugene) at 37°C for 10 min. MSR fluorescence was measured by flow cytometry (FACS Verse™, BD Biosciences, USA). Meanwhile, the ROS fluorescence intensity in the sperm treated with hydrogen peroxide was observed under a microscope.

### 2.3. Sperm RNA Extraction

Sperm sample collection and RNA extraction were performed as previously described [29]. The sperm were treated on ice with somatic cell lysis buffer for 40 min to eliminate somatic cell contamination. After removal of suspension, the sperm pellets were washed twice with PBS and pelleted at 600 g for 5 min. The sperm pellets were added with TRIzol reagent (Invitrogen, cat. no. 15596026) for RNA extraction.

### 2.4. Deep Sequencing and Small RNA Annotation

Small RNA libraries were performed as previously described [29]. All RNA library preparation and quality examination were performed by BGI. Small RNA sequences were annotated using the pipeline SPORTS (small noncoding RNA annotation Pipeline Optimized for and tsRNA rRNA [41]. Small RNA tags were annotated with miRNA, tRNA, rRNA, and other sncRNAs from miRBase19, Genbank, and Rfam databases using blastn with standard parameters: -F F -e 0.01. To analyze differential expression of small RNAs between H_2_O_2_-treated and control sperm, tsRNA and rsRNA reads were normalized to RPM (reads per million), respectively. The *P* value and *q* value between samples were generated by DEGseq package of R. Those small RNAs that had *P* value smaller than 0.05 and had the fold change number larger than 2 were labeled as significantly changed RNAs.

### 2.5. Northern Blots

Northern blot analysis was carried out as previously described [29]. RNA was separated by 15% urea-PAGE gel stained with SYBR Gold, immediately imaged, and then transferred to positively charged nylon membranes (GE Amersham, cat. no. MRPN303B). Membranes were prehybridized with DIG Easy Hyb solution (Roche; 11603558001) at 42°C for least 1 h. The membranes were immersed in probes which replenish with hybridization solution at a final concentration of 16 nM overnight (12-16 h) at 42°C. DIG-labeled oligonucleotide probes were synthesized by GENEWIZ, Inc. as the sequence as CCACTAGACCACCAGGGA for 5′-tsRNA-Glu.

### 2.6. Isolation of 29-34 nt RNAs from Sperm Total RNAs

Small RNAs sized at 29-34 nt were excised from the gel as previously described [5, 6]. One microgram of total sperm RNAs was separated by denatured 15% PAGE with 8 M urea. The gel was dyed with SYBR Green II stain stock solution (Invitrogen). The location of RNA fragments was determined by the standard small RNA markers with using long-wave UV light illumination of the gel.

### 2.7. Oocyte Collection, Zygote RNA Microinjection, and Embryo Transfer

Embryo collection and transfer were performed as previously described [29]. Intracytoplasmic sperm injection (ICSI) with MII (first polar body present) oocytes revealed two protoplasts and confirmed successful fertilization. The 29-34 nt RNAs isolated from OS or control sperm and chemically synthetic 5′ end phosphorylated tsRNA sequences (synthetic RNAs) or synthetic scrambled RNA (Table S1), with a concentration of 2 ng/*μ*L, were microinjected into the male pronuclei of fertilized eggs as previously described [5, 6]. The zygotes were then transferred to surrogate mother of C57BL/6 background.

### 2.8. Behavioral Tests

The offspring were tested on open field test at age of 7 weeks and light-dark box at age of 8 weeks for anxiety-like behavior and the forced swimming test at age of 9 weeks for depressive-like behavior. The same mice were used for all the experiments.

#### 2.8.1. Forced Swimming Test

The forced swimming test was conducted following the method described previously [42]. Briefly, mice were individually forced to swim in an open container at 25 ± 1°C. Latency to first immobility and total immobility time was manually scored during a 6 min period. The duration of immobility swimming time was recorded.

#### 2.8.2. Open Field Test

Open field box was made of cardboard with a center zone in the middle of the box marked with permanent marker and was used to analyze anxiety and locomotor activity [43]. Mice were placed near the wall of the arena and allowed to freely explore for 10 min while being recorded from a top mounted camera. Duration in the center was expressed as a percentage of the test duration.

#### 2.8.3. Light-Dark Box Test

The light-dark box test apparatus comprised an open-topped arena, one-third painted black and two-thirds white. The two compartments were separated by a wooden partition which had a small opening cut into its center at floor level. The time to exit from the dark to the light compartment and the percent of total time spent in the light compartment were measured. The natural propensity of mice to hide in the dark compartment is balanced by the animal exploratory behavior.

### 2.9. Serum Corticosterone Analysis

Serum corticosterone levels were quantified using an ELISA according to the manufacturer's instructions by Mouse Corticosterone ELISA Kit (E-EL-0161c, Elabscience Biotechnology Co. Ltd, Wuhan, China). The experimental procedures were as described in the manufacturer's instructions.

### 2.10. RT-PCR

Total RNA was extracted from hippocampus or liver using TRIzol reagent was performed as previously described [29]. One microgram of RNA was reverse transcribed using the M-MuLV Reverse Transcriptase Reaction system (NEB, cat. no. M0253L). Gene-specific primers were used with SYBR green (Promega, cat. no. A6002) for detection on a LightCycler 480 system (Roche). The primer sequences used were synthesized by BGI as shown Table S2.

### 2.11. Metabolic Testing

Mice were fasted overnight (16 h) before glucose tolerance test (GTT) or pyruvate tolerance test (PTT). The mice received intraperitoneal injection of 2 g glucose/kg body weight for GTT or 1.5 g pyruvate/kg body weight for PTT experiment. Blood collected from the tail was used to measure blood glucose levels at baseline (0), 15, 30, 60, 90, and 120 min after glucose injection by a glucose meter (ACCU-CHEK Active Blood Glucose Meter, Roche).

### 2.12. RNA-seq

Total RNA was extracted from liver of F1 male mice for RNA-seq as previously described [29]. The libraries were sequenced on Illumina NovaSeq 6000 sequencer as paired-end 150 bp reads following Illumina's instructions. Quantification of gene expression was performed using HTSeq v0.6.1 and gene annotations from Ensembl release. Only the Pearson correlation coefficient of interacted DEGs were greater than 0.997 are shown in this figure. The larger the circle, the greater the number of genes interacted with it.

### 2.13. Statistics

All data are presented as mean ± SEM and were analyzed with GraphPad Prism 7. Two-way ANOVA with uncorrected Fisher's LSD was used for GTT and PTT, and two-tailed unpaired Student's *t*-test was used for behavior test, AUC of GTT and PTT, levels of glucose and TG, and qPCR data. For each variable, Kolmogorov-Smirnov test was used to evaluate the normal distribution of values. The differences were considered statistically significant when *P* < 0.05.

## 3. Results

### 3.1. ROS-Induced Alterations of sncRNA Profiles in Sperm

**Figure 1:**
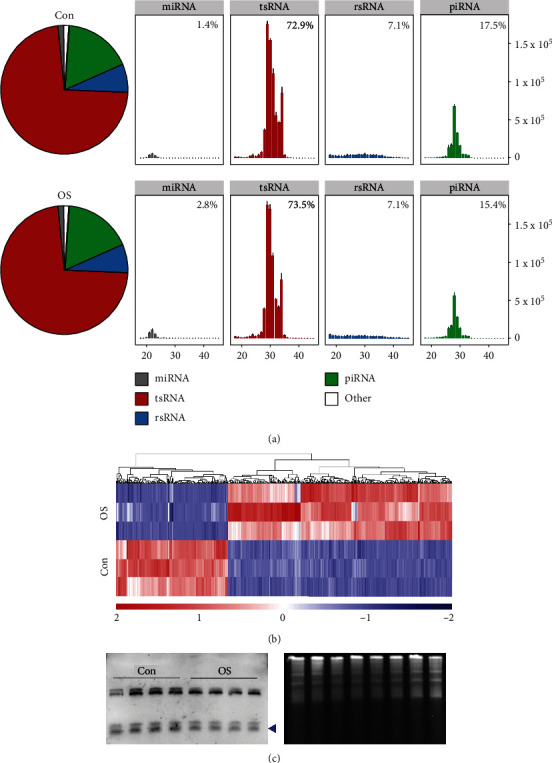
Composition of sncRNAs in sperm with or without oxidative stress. (a) Subcellular fractionation of miRNAs, tsRNAs, rsRNAs, and piRNAs in Con and OS sperm. (b) Heat map of differentially expressed 29-34 nt 5′-tsRNAs in sperm. (c) Northern blot analysis of 5′-tsRNA-Glu (shown by arrowheads) in sperm.

Sperm was collected and then incubated with 1500 *μ*M H_2_O_2_ for 3 h, as described previously [24]. The H_2_O_2_ treatment-induced OS caused the intracellular ROS levels in sperm to increase (Figure S1). To explore whether the sperm sncRNA profiles are affected by OS, we performed deep sequencing of sperm small RNAs. The results showed that OS could not cause significant changes in the proportion of tsRNAs and rsRNAs in sperm (Figure 1(a)). Because the 29-34 nt sncRNAs are highly expressed in mature mouse sperm [44], we further analyzed these RNAs in more detail (fold change > 2 and *P* < 0.05). Among all the 29-34 nt 5′-tsRNAs, the expression levels of 196 5′-tsRNAs increased whereas those of 394 5′-tsRNAs decreased in OS sperm (Figure 1(b), Figure S2, Table S3). The abundance of 5′-tsRNA-Glu in sperm was analyzed by Northern blot analysis (Figure 1(c)). Among all the 29-34 nt rsRNAs, the expression levels of 6 rsRNAs increased whereas those of 70 rsRNAs decreased in OS sperm. These data reveal that ROS could induce changes of sncRNA profiles in sperm.

### 3.2. Injection of Sperm RNA Fragment Induces Depressive-Like and Anxiety-Like Behaviors in Adult Male Offspring

**Figure 2:**
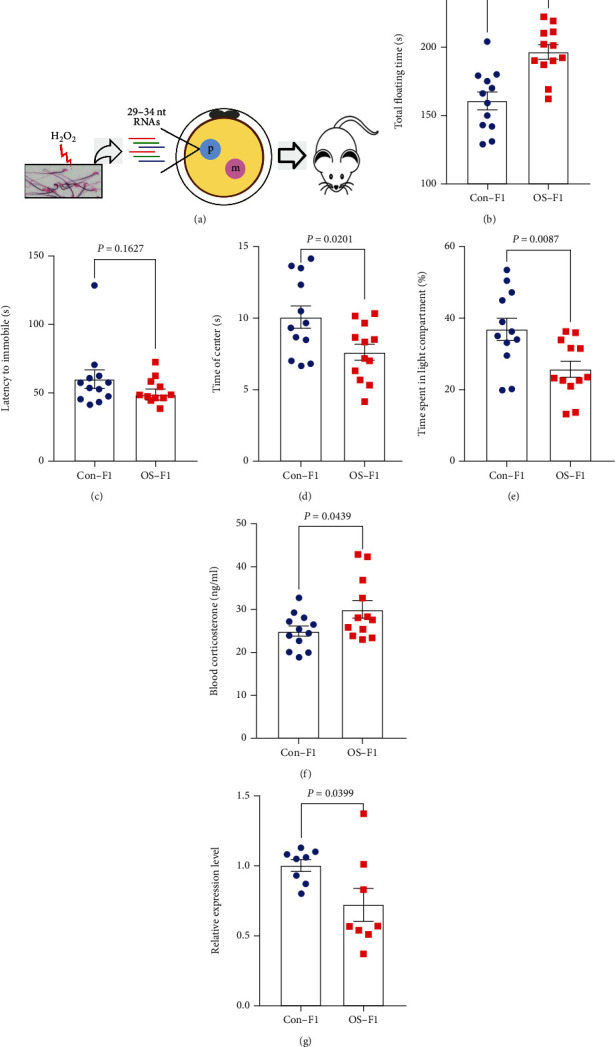
Behavior, blood corticosterone, and hippocampal GR in male offspring generated from sperm 29-34 nt RNA injection. (a) Illustration of zygotic injection of sperm 29-34 nt RNAs to generate F1 male offspring. (b) Forced swimming test. (c) Latency to first immobility of forced swimming test. (d) Open field test. (e) Light-dark box test. (f) Blood concentration of corticosterone detected by ELISA. In (b–f), *n* = 12 mice per group. (g) The relative expression level of *GR* mRNA in hippocampus detected by RT-PCR. *n* = 8 mice per group. All data are plotted as means ± SEM. Each dot represents one mouse.

The 29-34 nt RNA fragments were collected from control and OS sperm and injected into the male pronuclei of normal zygote following a previously established zygotic RNA injection protocol (Figure 2(a)) [5, 6]. We found that the RNA injection had no adverse effects on the embryo development (Table S4). The body weight and body composition of the resultant offspring from control (Con-F1) and oxidative stress (OS-F1) sperm RNA fragment injection have shown no significantly difference (Table S5 and Table S6). The depressive-like and anxiety-like behaviors of the offspring were tested. In the forced swimming test, the immobility time of OS-F1 male mice was longer compared with that of Con-F1 male mice (Figure 2(b), *P* = 0.0008; 155.3 ± 3.9  s vs. 129.0 ± 5.5 ), and there was no significant difference in latency to immobility which suggests that it exhibited depressive-like behavior in male mice (Figure 2(c)). In the open field test, the time of OS-F1 male mice spent in the center areas was significantly less than that of Con-F1 (Figure 2(d), *P* = 0.0201; 7.6 ± 0.6% vs. 10.1 ± 0.8%). In the light-dark box test, the time spent in the light compartment of OS-F1 mice was less than that of Con-F1 (Figure 2(e), *P* = 0.0087; 25.8 ± 2.3% vs. 37.0 ± 3.1%). However, there was no significant difference in total floating time (Figure S3a) and immobility time (Figure S3b) during forced swimming test in female mice. Also, there was no significant difference in time spent in the center areas (Figure S3c) during open field test and time spent in the light (Figure S3d) during the light-dark box test in female mice. Taken together, these results suggest that OS-F1 male but not female mice exhibited anxiety-like behavior.

Corticosterone in plasma plays a key role in the hypothalamic-pituitary-adrenal (HPA) axis could be a well characterized marker of stress intensity [45], wherein OS-F1 mice were more markedly elevated than those in Con-F1 male mice (Figure 2(f)). Moreover, the level of glucocorticoid receptor (*GR*) mRNA in hippocampus in OS-F1 male mice was lower than that in Con-F1 mice (Figure 2(g)).

### 3.3. Injection of Synthetic RNAs Induces Depressive-Like and Anxiety-Like Behaviors in Adult Male Offspring

**Figure 3:**
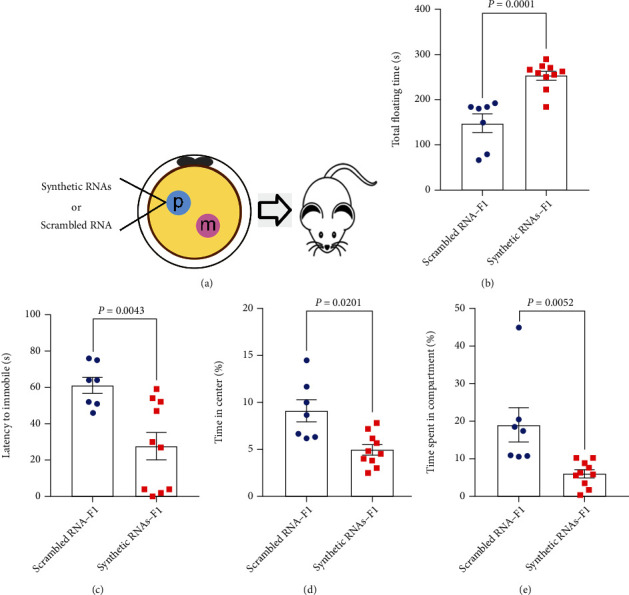
Behavior in male offspring generated from synthetic RNA injection. (a) Illustration of zygotic injection of synthetic tsRNAs to generate F1 male offspring. (b) Forced swimming test. (c) Latency to first immobility of forced swimming test. (d) Open field test. (e) Light-dark box test. *n* = 7 mice in scrambled RNA group and *n* = 10 mice in synthetic RNA group. All data are plotted as means ± SEM. Each dot represents one mouse.

We next synthesized the most highly expressed 5′-tsRNAs which are abundant in the OS sperm and injected into the male pronuclei of normal zygote to investigate whether they could mimic the function of endogenous sperm sncRNAs (Figure 3(a)). In the forced swimming test, the immobility time of synthetic RNAs-F1 male mice were longer compared with that of scrambled RNA-F1 male mice (Figure 3(b), *P* = 0.0001; 253.1 ± 9.5 s vs. 147.7 ± 20.1 s). Also, the immobility time of synthetic RNAs-F1 female mice were longer compared with that of scrambled RNA-F1 female mice (Figure S4a, *P* = 0.0265; 249.7 ± 12.0 s vs. 185.9 ± 17.8 s). The time of latency to immobility of synthetic RNAs-F1 male mice was less than that of scrambled RNA-F1 male mice (Figure 3(c), *P* = 0.0043; 27.9 ± 7.6  s vs. 61.1 ± 4.9) but not female mice (Figure S4b). In the open field test, the time of synthetic RNAs-F1 male mice spent in the center areas was significantly less than that of scrambled RNA-F1 (Figure 3(d), *P* = 0.0029; 5.0 ± 0.1% vs. 9.1 ± 1.1%) and female mice (Figure S4c, *P* = 0.0025; 31.7 ± 4.5% vs. 57.5 ± 4.6%). In the light-dark box test, the time spent in the light compartment of synthetic RNAs-F1 male mice was less than that of scrambled RNA-F1 mice (Figure 3(e), *P* = 0.0052; 6.1 ± 1.1% vs. 19.1 ± 4.6%) and also in female mice (Figure S4d, *P* = 0.0052; 6.1 ± 1.1% vs. 19.1 ± 4.6%). Taken together, these results show that zygotic injection of a pull of synthetic tsRNAs can partly induce depressive-like and anxiety-like behaviors in offspring.

### 3.4. Injection of Sperm RNA Fragments Alters Hepatic Metabolic Phenotypes of Adult Male Offspring

**Figure 4:**
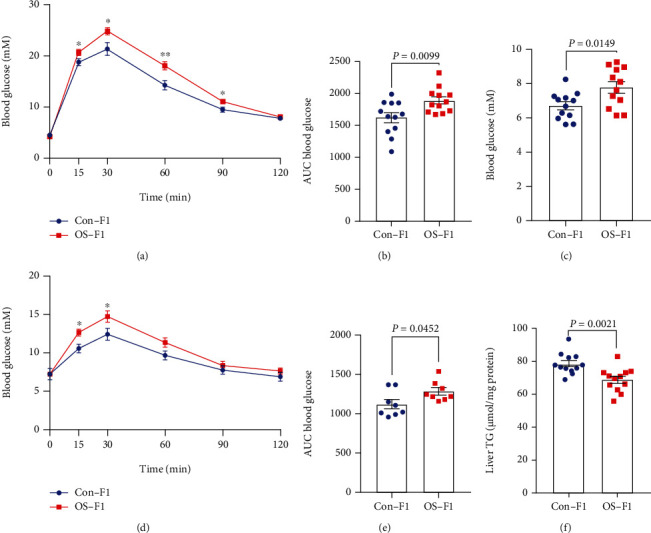
Metabolic parameters in male offspring generated from sperm 29-34 nt RNA injection. (a) Blood glucose during GTT in F1 males. (b) The area under curve (AUC) statistics for GTT. ^∗^*P* < 0.05; ^∗∗^*P* < 0.01. (c) Blood glucose in fasting conditions. (d) Blood glucose during PTT in F1 males. (e) The AUC statistics for PTT. ^∗^*P* < 0.05. (f) The content of TG in liver. *n* = 12 mice per group. All data are plotted as means ± SEM. Each dot represents one mouse.

OS-F1 male mice maintained on a chow diet developed impaired glucose tolerance. The blood glucose levels during GTT were significantly higher than those of Con-F1 mice at 14 weeks of age (Figures 4(a) and 4(b)). Additionally, compared with Con-F1 mice, OS-F1 male mice had elevated blood glucose levels during fasting (Figure 4(c)) and had higher glucose levels during PTT, an indication of hyperactive hepatic gluconeogenesis (Figures 4(d) and 4(e)). However, there was no significant difference in blood glucose levels during GTT (Figure S5a-b) and blood glucose levels during fasting (Figure S5c). Moreover, the content of triglyceride in the liver of OS-F1 mice was lower than that of Con-F1 mice (Figure 4(f)). Overall, these results demonstrate that hepatic gluconeogenesis of mice in the OS-F1 male mice was elevated.

### 3.5. Injection of Sperm RNA Fragments Alters Hepatic Gene Expression of Adult Male Offspring

**Figure 5:**
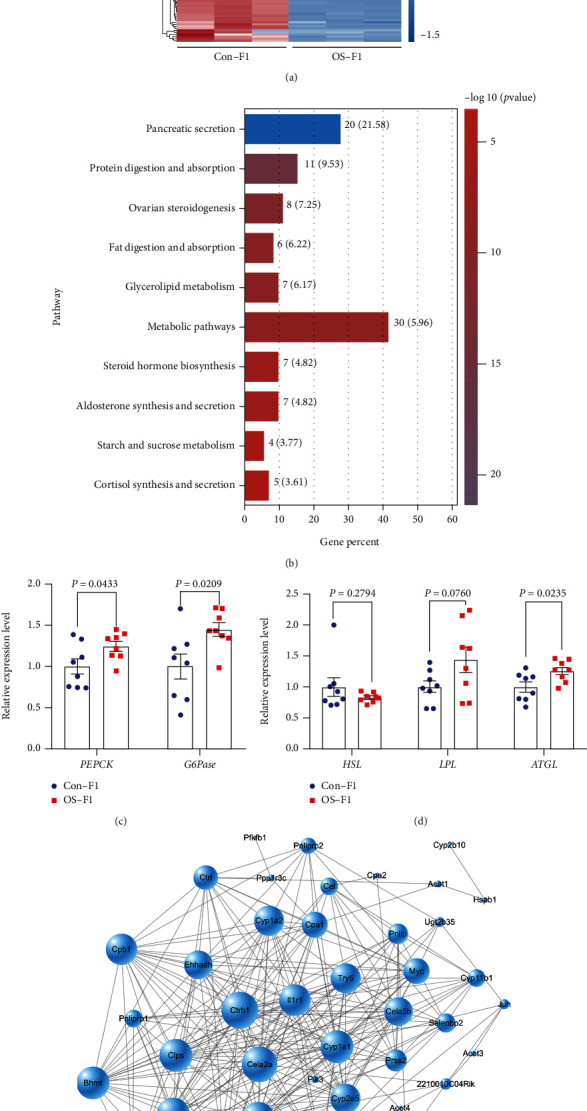
The hepatic gene expression in male offspring generated from sperm 29-34 nt RNA injection. (a) Expression levels of genes affected by sperm 29-34 nt RNA injection visualized by heat map in liver samples of F1 male offspring. (b) The top ten significant clusters of gene ontology (KEGG) terms enriched in liver samples of F1 male offspring determined by GSEA and clustered under parent terms were related to synaptic signaling (*n* = 3, NES > |2| and FDR < 0.05). (c) The relative expression levels of gluconeogenic genes *PEPCK* and *G6Pase* in the liver of F1 male mice. (d) The relative expression levels of lipolytic genes *HSL*, *LPL*, and *ATGL* in the liver of F1 male mice. *n* = 8 mice per group. All data are plotted as means ± SEM, and each dot represents one mouse. (e) Protein-protein interaction networks of DEGs in the partially top 20 enriched KEGG pathways related to metabolism.

To obtain an overall view of the transcriptional response, RNA purified from liver samples of OS-F1 and Con-F1 male mice at 17 weeks of age was sequenced. A total of 129 genes (68 upregulated genes and 61 downregulated genes) were found to be differentially expressed (Figure 5(a)). Pathway analysis of these differentially expressed genes showed that these genes were enriched in the metabolic pathways (fold change > 2 and *P* < 0.05) (Figure 5(b)). The expression level of gluconeogenic (*PEPCK* and *G6Pase*) (Figure 5(c)) and lipolytic (*ATGL*) genes was higher in the liver of OS-F1 male mice than that of the Con-F1 mice (Figure 5(d)). The protein-protein interaction networks of DEGs in the partially top 20 enriched KEGG pathways related to metabolism (Figure 5(e)). *Ctrb1* emerged as a hub protein in the network, linking with several metabolic pathway-related genes, such as *Cela2a*, *Cyp2c70*, *Cyp1a1*, and *Cyp2a5* (Figure 5(e)). Altogether, male offspring maintained on a chow diet developed impaired glucose tolerance and hyperactive hepatic gluconeogenesis, accompanied by the upregulation of hepatic gluconeogenic and lipolytic genes.

## 4. Discussion

**Figure 6:**
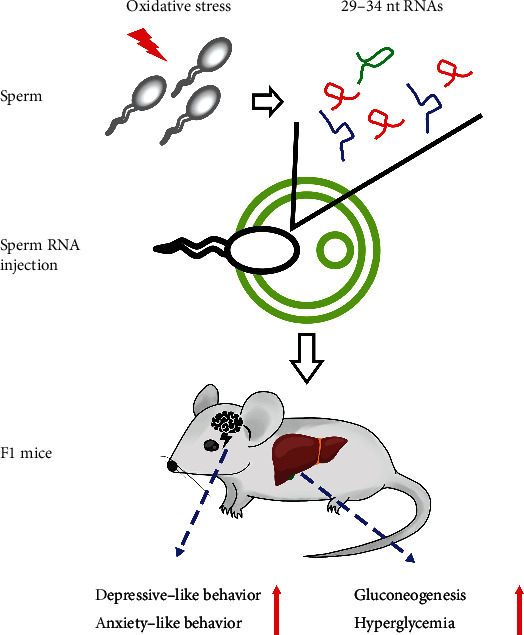
A schematic representing the role of sncRNAs in sperm oxidative stress-induced programming of behavioral and metabolic phenotypes in offspring.

Increasing evidence has indicated that paternal environmental inputs can induce alterations of behavioral and metabolic phenotypes of the next generation; however, little is known about the mechanisms involved in this process. Here, we demonstrated that ROS could induce the changes in sperm sncRNA profiles. Zygotic injection of 29-34 nt RNA fragments from OS sperm or a pull of synthetic tsRNAs could induce depressive-like and anxiety-like behaviors and glucose intolerance in male but not female offspring. It suggests that ROS-induced change of sperm sncRNA profiles contributes to the alterations of behavioral and metabolic phenotypes of the offspring (Figure 6).

Sperm sncRNA contributes to the intergenerational transmission as a potential carrier [5, 25, 29, 31]. A large amount of researches show that sncRNAs mediate the transmission of paternal traits to offspring is based on long-term exposure from testis to epididymis [5, 6, 25, 32, 33]. It has been reported that tRNA cleavage was by several classes of self-cleaving ribozymes including RNase A, tRNA splicing endonuclease, RNase T2, and RNase L into tsRNAs [46]. There are several RNases expressed in male germ cell [47, 48] and mature sperm [49, 50] and is essential for mammalian spermatogenesis and male fertility. Recently, research has indicated that ROS can mediate the cleavage of tRNAs into tsRNAs and the cleavage of rRNAs into rsRNAs by regulating their biogenesis pathways [38]. The exposed sites of the tRNA structure could be “points of attack” in an ancient cellular environment, being fragmented by either nonspecific stress signals such as ROS, specific recognition by enzymes, or ribozymes [46]. In the present study, ROS induced changes in mature sperm sncRNA profiles. Also, Dai et al. reported that the abundance of mRNAs and miRNAs was altered during cryopreservation by sperm exposure to oxidative stress [51]. These results suggested that ROS induced small RNA processing in mature sperm. Further studies are required in order to better understand how ROS mediate the cleavage of tRNA into tsRNAs and rRNA cleavage into rsRNA in mature sperm.

Increasing evidence now suggests that sperm sncRNAs can mediate intergenerational effects but the underlying processes and mechanisms remain puzzling. It has been reported that transfection of sncRNAs promoted lineage differentiation in embryoid bodies and embryonic stem cells in mice [52, 53]. The alteration in the early embryo might trigger a chain reaction that continuously influences the metabolic state in the offspring [11]. Gluconeogenesis is primarily modulated by PEPCK and G6Pase [54]. In the present study, we found that liver tissue of OS-F1 mice showed a significant increase in *PEPCK* and *G6Pase*. Interestingly, a recent study found that 5′-tsRNA-Gly-GCC that increased in HFD mice mature sperms can promote gluconeogenesis in liver by regulating Sirt6-FoxO1 pathway, which activates PEPCK and G6Pase [55]. Thus, the phenotypic outcome of injecting 29-34 nt RNA fragments from OS sperm may relate to the function of sncRNAs in cell fate regulation in the early embryo and continuously influences the metabolic state in the offspring. Further work is needed to elucidate the function of sperm sncRNAs in association with tsRNA-mediated intergenerational effects.

It has been demonstrated that *in vitro* fertilization (IVF) may affect embryo development, causing multiple adverse effects on health during the postnatal life of the embryo, contributing to the development of chronic adult-onset diseases, such as type 2 diabetes, metabolic syndrome, and cardiovascular disease [56–58]. During IVF, the level of ROS in sperm could arise from several exogenous factors such as exposure to visible light, cryopreservation, O_2_ tension, and temperature [21, 59]. In this study, one important observation was that sncRNA profiles in sperm could rapidly response to the presence of ROS. Recently, several studies have indicated that sperm tsRNA profiles may be used as a potential biomarker for evaluating male fertility during IVF [60, 61]. Our results indicated that OS-induced alteration of sncRNAs in sperm might be involved in the fetal origins of adult diseases. This information may be used as a guideline for sperm preservation, artificial insemination, and IVF, as well as for precision medicine.

Taken together, our data showed a role on ROS in “sperm RNA code” and phenotypes of offspring as follows: (i) ROS could alter the sncRNA profiles in sperm; (ii) sncRNAs were essential for ROS in sperm to induce the programing of behavioral and metabolic phenotypes of the offspring. Further exploration of these mechanisms is required in order to gain deeper insights into the developmental programming of health and disease of the offspring based on the paternal health at the time of conception.

## Data Availability

The small RNA-seq and transcriptome sequencing data generated in this study have been deposited in the NCBI Sequence Read Archive (SRA) database under the BioProject accession number PRJNA698641. All data supporting the findings of this study are available from the corresponding author on reasonable request.

## Supplementary Materials

Supplementary Materials Supplementary Figure 1: The content of ROS in sperm with or without H_2_O_2_. Supplementary Figure 2: Locus mapping information of tsRNAs in different tRNAs for Con and OS sperm. Supplementary Figure 3: Behavior in female offspring generated from sperm 29-34 nt RNA injection. Supplementary Figure 4: Behavior in female offspring generated from synthetic RNA injection. Supplementary Figure 5: Metabolic parameters in female offspring generated from synthetic RNA injection. Supplementary Table 1: Sequences of synthetic tsRNAs and scrambled RNA injected into normal zygotes. Supplementary Table 2: Nucleotide sequences of specific primers for qRT-PCR. Supplementary Table 3: Abundance of sperm 5′-tsRNAs in Con and OS sperm. Supplementary Table 4: Summary of outcome after sperm 30-40 nt RNAs and water injection into normal zygotes. Supplementary Table 5: Body composition and fasting blood glucose in F1 male mice at 17 weeks of age. Supplementary Table 6: Body composition and fasting blood glucose in F1 female mice at 17 weeks of age.
