# Supplementary material for: Small Noncoding RNAs Contribute to Sperm Oxidative Stress-Induced Programming of Behavioral and Metabolic Phenotypes in Offspring
Source: Oxid Med Cell Longev. 2022 Jun 6;2022:6877283. doi: 10.1155/2022/6877283 (PMC9192199; doi:10.1155/2022/6877283)
Supplement: Supplementary Materials — Supplementary Figure 1: The content of ROS in sperm with or without H2O2. Supplementary Figure 2: Locus mapping information of tsRNAs in different tRNAs for Con and OS sperm. Supplementary Figure 3: Behavior in female offspring generated from sperm 29-34 nt RNA injection. Supplementary Figure 4: Behavior in female offspring generated from synthetic RNA injection. Supplementary Figure 5: Metabolic parameters in female offspring generated from synthetic RNA injection. Supplementary Table 1: Sequences of synthetic tsRNAs and scrambled RNA injected into normal zygotes. Supplementary Table 2: Nucleotide sequences of specific primers for qRT-PCR. Supplementary Table 3: Abundance of sperm 5′-tsRNAs in Con and OS sperm. Supplementary Table 4: Summary of outcome after sperm 30-40 nt RNAs and water injection into normal zygotes. Supplementary Table 5: Body composition and fasting blood glucose in F1 male mice at 17 weeks of age. Supplementary Table 6: Body composition and fasting blood glucose in F1 female mice at 17 weeks of age. [file 6877283.f1.docx]

Supplementary Information

**Small non-coding RNAs contribute to sperm oxidative stress-induced programming of behavioral and metabolic phenotypes in offspring**

Li Ren, Yining Xin, Xiaoxiao Sun, Yanwen Zhang, Yingqi Chen, Suyuan Liu, Bin He *

*Corresponding author

E-mail: [heb@njau.edu.cn](mailto:heb@njau.edu.cn)

This PDF file includes:

Supplementary Figures 1 to 5

Supplementary Tables 1 to 6


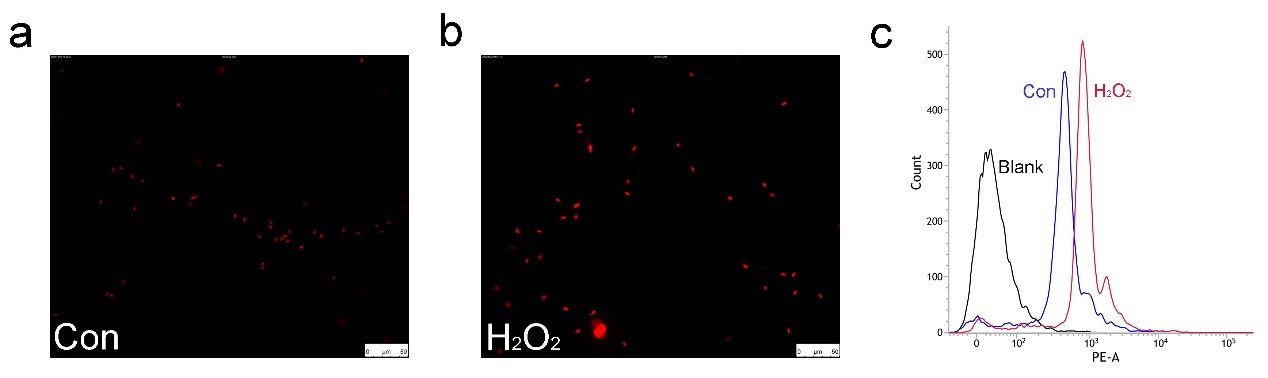


**Supplementary Figure 1 The content of ROS in sperm with or without H_2_O_2_.**

**a** Measuring ROS content in sperm using MitoSOX staining and detected by fluorescence microscope. **b** Measuring ROS content in sperm using MitoSOX staining and flow cytometry analysis.


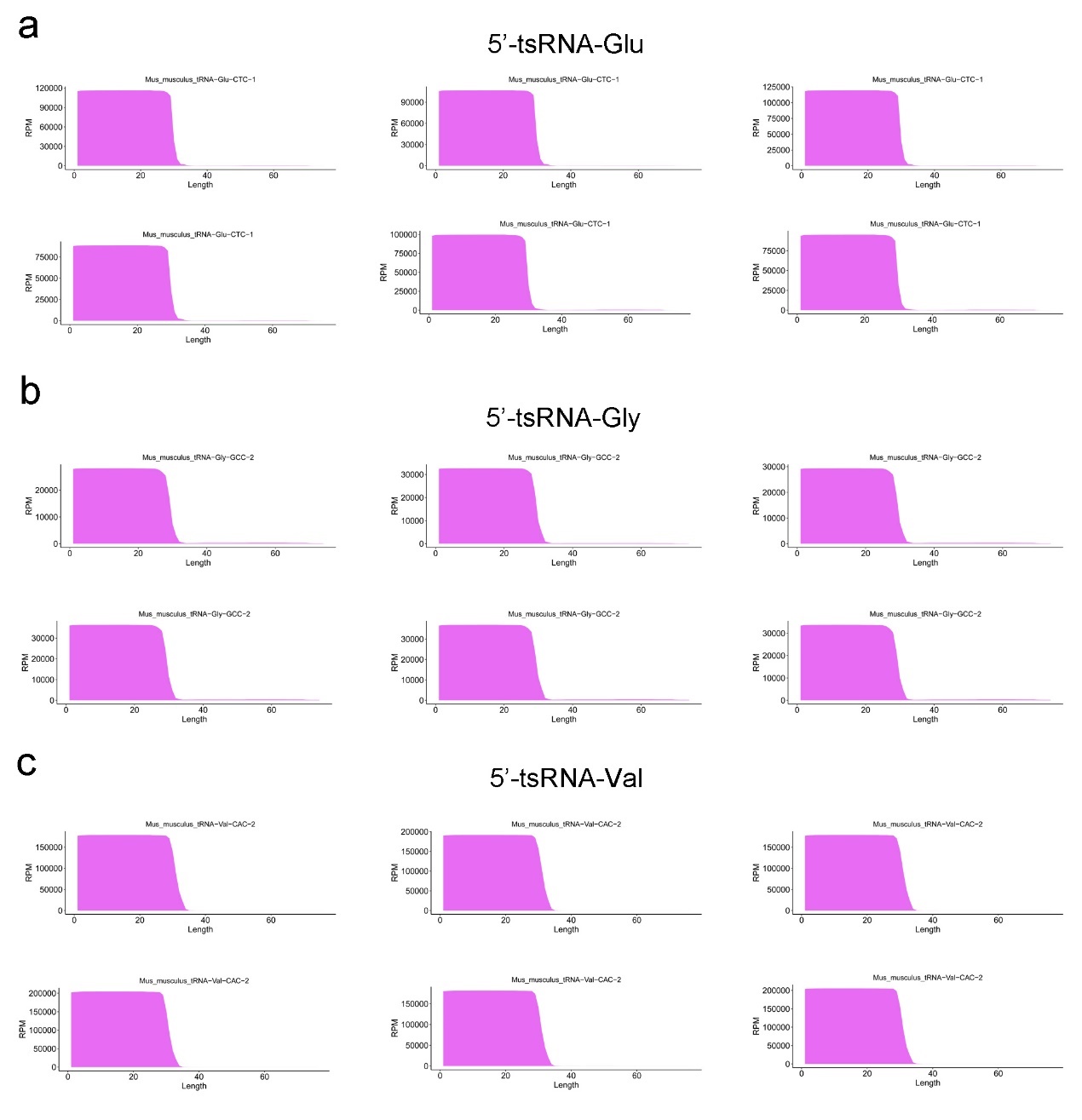


**Supplementary Figure 2 Loci mapping information of tsRNAs in different tRNAs for Con and OS sperm.**

RPM: reads per million.


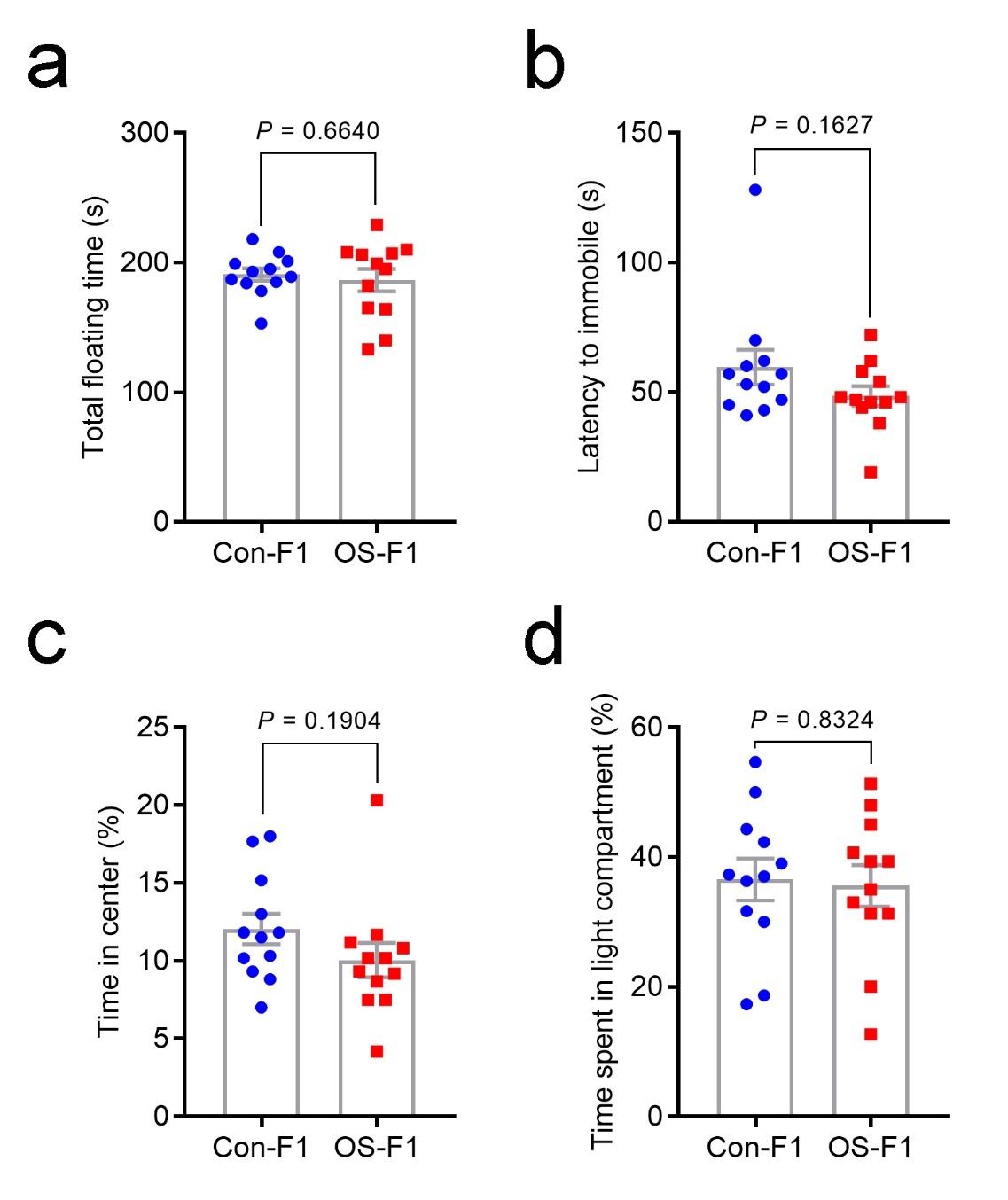


**Supplementary Figure 3 Behavior in female offspring generated from sperm 29-34 nt RNAs injection.**

**a** Forced swimming test. **b** Latency to first immobility of forced swimming test. **c** Open field test. **d** Light-dark box test. n = 12 mice per group. All data are plotted as means ± s.e.m. Each dot represents one mouse.


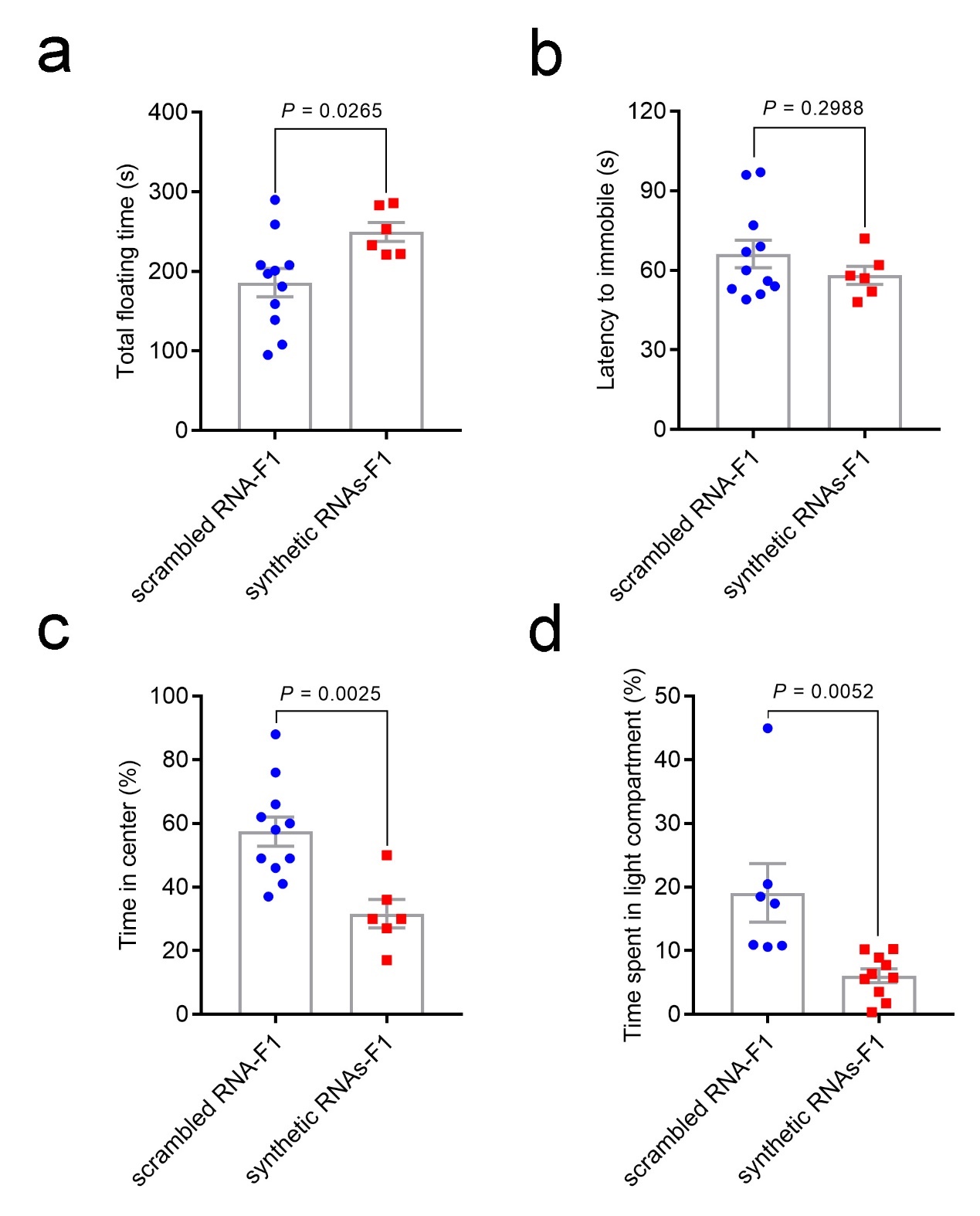


**Supplementary Figure 4 Behavior in female offspring generated from synthetic RNAs injection.**

**a** Forced swimming tests. **b** Latency to first immobility of forced swimming test. **c** Open field test. **d** Light-dark box test. n = 11 mice in scrambled RNA group and n = 6 mice in synthetic RNAs group. All data are plotted as means ± s.e.m. Each dot represents one mouse.


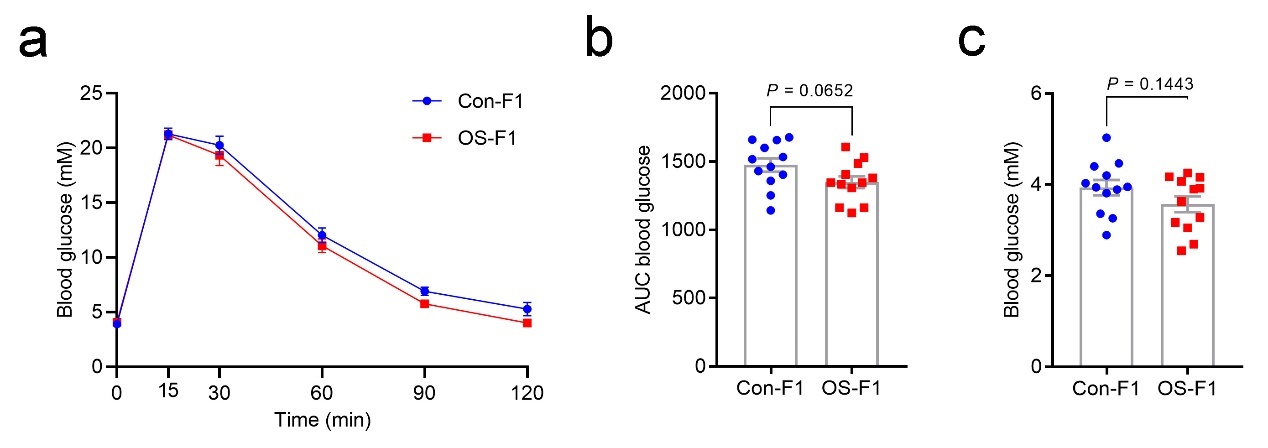


**Supplementary Figure 5 Metabolic parameters in female offspring generated from synthetic RNAs injection.**

**a** Blood glucose during GTT in F1 females. **b** The AUC statistics for GTT. **c** Blood glucose in fasting conditions in F1 males. n = 12 mice per group. All data are plotted as means ± s.e.m. Each dot represents one mouse.

**Supplementary Table 1** Sequences of synthetic tsRNAs and scrambled RNA injected into normal zygotes.

| tsRNA | Sequences (5’ to 3’) |
| --- | --- |
| tsRNA-Glu-CTC; tsRNA-Glu-TTC | UCCCUGGUGGUCUAGUGGUUAGGAUUCGGCG |
| tsRNA-Glu-TTC | UCCCACAUGGUCUAGCGGUUAGGAUUCCUGGUU |
| tsRNA-Gly-CCC | GCGCCGCUGGUGUAGUGGUAUCAUGCAAGAUUC |
| tsRNA-Gly-GCC | GCAUUUGUGGUUCAGUGGUAGAAUUCUCGCC |
| tsRNA-Gly-GCC; tsRNA-Gly-CCC | GCAUUGGUGGUUCAGUGGUAGAAUUCUCGCC |
| tsRNA-Gly-GCC; tsRNA-Gly-CCC | GCAUUGGUGGUUCAGUGGUAGAAUUCUCGC |
| tsRNA-iMet-CAT | AGCAGAGUGGCGCAGCGGAAGCGUGCUGGGCCC |
| tsRNA-Val-CAC; tsRNA-Val-AAC | GUUUCCGUAGUGUAGUGGUUAUCACGUUCGCC |
| tsRNA-Val-TAC | GGUUCCAUAGUGUAGCGGUUAUCACGUCUGCUUU |
| tsRNA-Val-TAC | GGUUCCAUAGUGUAGCGGUUAUCACGUCUGCUU |
| Scrambled RNA | CCUCCCAAAGUGCUGGGAUUACAGGCGUGAG |

**Supplementary Table 2** Nucleotide sequences of specific primers for qRT-PCR.

| Target genes | Sequences (5’ to 3’) | GenBank No. |
| --- | --- | --- |
| *GR* | F: CAAGGGTCTGGAGAGGACAA | NM_001361209.1 |
|  | R: TACAGCTTCCACACGTCAGC |  |
| *PEPCK* | F: GACCATAACATAGTATACACCTGCTGC | NM_011044 |
|  | R: AGAAGGGTCGCATGGCAA |  |
| *G6Pase* | F: CACCTGTGAGACCGGACCA | NM_008061 |
|  | R: GACCATAACATAGTATACACCTGCTGC |  |
| *HSL* | F: CTCACAGTTACCATCTCACCTC | NM_001039507 |
|  | R: GATTTTGCCAGGCTGTTGAGTA |  |
| *ATGL* | F: CAGAGATGGACTTCGATTCCTT | NM_025802 |
|  | R: CAGGTGCTCTAGAATTCGATCT |  |
| *LPL* | F: GCGTAGTTCCAGCAGCAAAG | NM_008509 |
|  | R: AGAAATCTCTTCCCGCGTCTG |  |
| *GAPDH* | F: TCTCCTGCGACTTCAACA | GU214026.1 |
|  | R: TGTAGCCGTATTCATTGTCA |  |

**Supplementary Table 3** Abundance of sperm 5'-tsRNAs in Con and OS sperm.

| 5'-tsRNA | Sequence | *P* value |
| --- | --- | --- |
| mature-mt_tRNA-Gly-TCC_5_end | ACTCCCTTGGTATAATTAATATAACTGACT | 0.0349247 |
| mature-mt_tRNA-Met-CAT_5_end | AGTAAGGTCAGCTAATTAAGCTATCGGGCC | 0 |
| mature-mt_tRNA-Met-CAT_5_end | AGTAAGGTCAGCTAATTAAGCTATCGGGC | 1.74E-05 |
| mature-tRNA-Ala-CGC_5_end | ACCCTGGTAGTCTAGTGGTTAGGATTCGG | 0.0067401 |
| mature-tRNA-Asp-GTC_5_end | TCCTCGTTAGTGTAGTGGTGAGTATCCCCGCCTG | 0.0466795 |
| mature-tRNA-Asp-GTC_5_end | GCCTCGTTAGTATAGTGGTGAGTATCCCCGCCTG | 0.0466795 |
| mature-tRNA-Asp-GTC_5_end | ACCTCGTTAGTATAGTGGTGAGTATCCCCGCCTG | 1.00E-05 |
| mature-tRNA-Asp-GTC_5_end | ACCTCGTTAGTATAGTGGTTAGTATCCCCGCCTG | 0.0088667 |
| mature-tRNA-Asp-GTC_5_end | TCCTCGTTAGTATAGTGGTGAGTATCCCCCCCTG | 0.005808 |
| mature-tRNA-Asp-GTC_5_end | TCCTCGTCAGTATAGTGGTGAGTATCCCCGCCTG | 0.0157469 |
| mature-tRNA-Asp-GTC_5_end | TCCTCGTGAGTATAGTGGTGAGTATCCCCGCCTG | 0.001781 |
| mature-tRNA-Asp-GTC_5_end | TCCTCGTAAGTATAGTGGTGAGTATCCCCGCCTG | 0.0009195 |
| mature-tRNA-Gln-CTG_5_end | GGTTCCATGGTGTAATGGTTAGCACTCTGC | 0.0273475 |
| mature-tRNA-Gln-CTG_5_end | GGTTCCATGCTGTAATGGTTAGCACTCTGG | 0.0280349 |
| mature-tRNA-Gln-CTG_5_end | GGTTCCATGGTGGAATGGTTAGCACTCTGG | 0.0243621 |
| mature-tRNA-Gln-CTG_5_end | GGTTCCATGGTGAAATGGTTAGCACTCTGG | 0.0243621 |
| mature-tRNA-Glu-CTC_5_end | TCCCTGGTGGTATAGTGGTTAGGATTCGGCG | 0 |
| mature-tRNA-Glu-CTC_5_end | TCCCTGGTGGTATAGTGGTTAGGATTCGGC | 0 |
| mature-tRNA-Glu-CTC_5_end | TCCCTGGTGGTATAGTGGTTAGGATTCGG | 0 |
| mature-tRNA-Glu-CTC_5_end | TCCCTGGTGGTGTAGTGGTTAGGATTCGGCGCT | 0.0066972 |
| mature-tRNA-Glu-CTC_5_end | TCCCTGGTGGTGTAGTGGTTAGGATTCGGCG | 1.11E-16 |
| mature-tRNA-Glu-CTC_5_end | TCCCTGGTGGTGTAGTGGTTAGGATTCGG | 0 |
| mature-tRNA-Glu-CTC_5_end | TCCCTGGTGGTGTAGTGGTTAGGATTCGGC | 0 |
| mature-tRNA-Glu-CTC_5_end | TCCCTGGTGGTTTAGTGGTTAGGATTCGGCG | 2.76E-09 |
| mature-tRNA-Glu-CTC_5_end | TCCCTGGTGGTTTAGTGGTTAGGATTCGGCGCT | 0.0466795 |
| mature-tRNA-Glu-CTC_5_end | TCCCTGGTGGTTTAGTGGTTAGGATTCGGC | 0 |
| mature-tRNA-Glu-CTC_5_end | TCCCTGGTGGTTTAGTGGTTAGGATTCGG | 0 |
| mature-tRNA-Glu-CTC_5_end | TCGCTGGTGGTCTAGTGGTTAGGATTCGG | 3.37E-13 |
| mature-tRNA-Glu-CTC_5_end | TCGCTGGTGGTCTAGTGGTTAGGATTCGGCG | 0.0463513 |
| mature-tRNA-Glu-CTC_5_end | TCGCTGGTGGTCTAGTGGTTAGGATTCGGC | 4.94E-05 |
| mature-tRNA-Glu-CTC_5_end | TCCCTGGTGGGCTAGTGGTTAGGATTCGGC | 0.0032324 |
| mature-tRNA-Glu-CTC_5_end | TCCATGGTGGTCTAGTGGTTAGGATTCGG | 6.68E-10 |
| mature-tRNA-Glu-CTC_5_end | TCCATGGTGGTCTAGTGGTTAGGATTCGGC | 0.0002314 |
| mature-tRNA-Glu-CTC_5_end | TCCATGGTGGTCTAGTGGTTAGGATTCGGCG | 0.0384405 |
| mature-tRNA-Glu-CTC_5_end | TCCCTGGTGGGCTAGTGGTTAGGATTCGG | 1.09E-05 |
| mature-tRNA-Glu-CTC_5_end | ACCCTGGTGGTCTAGTGGTTAGGATTCGGCG | 2.68E-07 |
| mature-tRNA-Glu-CTC_5_end | TCCCTGGTGGTCTACTGGTTAGGATTCGG | 6.61E-09 |
| mature-tRNA-Glu-CTC_5_end | TCCCTGGTGGTCTAGAGGTTAGGATTCGG | 0.000957 |
| mature-tRNA-Glu-CTC_5_end | TCCCTGGTGGTCTAGTGGTTTGGATTCGG | 0.005108 |
| mature-tRNA-Glu-CTC_5_end | TCCCTGGTGGTCTACTGGTTAGGATTCGGCG | 0.0372006 |
| mature-tRNA-Glu-CTC_5_end | TCCCTGGTGGTCTAGTGGTTAGGATTCGGCGGT | 0.0003819 |
| mature-tRNA-Glu-CTC_5_end | TCCCTGGTGGTCTAGTGGTTAGCATTCGGC | 1.83E-07 |
| mature-tRNA-Glu-CTC_5_end | TCCCTGGTGGTCTAGTGGTTAAGATTCGG | 0.0082136 |
| mature-tRNA-Glu-CTC_5_end | TCCCTGGTGGTCTAGCGGTTAGGATTCGGC | 0.046645 |
| mature-tRNA-Glu-CTC_5_end | TCCCTGGTGTTCTAGTGGTTAGGATTCGGCG | 0.0250292 |
| mature-tRNA-Glu-CTC_5_end | TCCCTGGTGGTCTAGGGGTTAGGATTCGG | 4.75E-05 |
| mature-tRNA-Glu-CTC_5_end | TCCCTGGTGGTCTAGCGGTTAGGATTCGG | 0.0001038 |
| mature-tRNA-Glu-CTC_5_end | TCCCTGGTGGTCTAGTGGTTAAGATTCGGC | 0.011721 |
| mature-tRNA-Glu-CTC_5_end | TCCCTGGTGGTCTAGGGGTTAGGATTCGGC | 0.0250292 |
| mature-tRNA-Glu-CTC_5_end | TCCCTGGTGTTCTAGTGGTTAGGATTCGGC | 1.23E-05 |
| mature-tRNA-Glu-CTC_5_end | TCCCTGGTGGTCTAGTGGTTAGGATTCTGCG | 0.0105406 |
| mature-tRNA-Glu-CTC_5_end | TCCCTGGTGGTCTAGTGTTTAGGATTCGGC | 0 |
| mature-tRNA-Glu-CTC_5_end | TCCCTGGTGGTCTAGTGGTTAGGATTCGCCG | 2.69E-07 |
| mature-tRNA-Glu-CTC_5_end | TCCCTGGTGGTCAAGTGGTTAGGATTCGGC | 0.0121267 |
| mature-tRNA-Glu-CTC_5_end | TCCCTGGTGTTCTAGTGGTTAGGATTCGG | 2.38E-11 |
| mature-tRNA-Glu-CTC_5_end | TCCCTGGTGGTCTAGTGTTTAGGATTCGG | 0 |
| mature-tRNA-Glu-CTC_5_end | TCCCTCGTGGTCTAGTGGTTAGGATTCGGCG | 3.04E-06 |
| mature-tRNA-Glu-CTC_5_end | TCCCTCGTGGTCTAGTGGTTAGGATTCGG | 0 |
| mature-tRNA-Glu-CTC_5_end | TCCCTCGTGGTCTAGTGGTTAGGATTCGGC | 0 |
| mature-tRNA-Glu-CTC_5_end | TCCCTGGAGGTCTAGTGGTTAGGATTCGGC | 0 |
| mature-tRNA-Glu-CTC_5_end | TCCCTGGTGGTCTAGTGGTTAGGATTCCGCGCT | 0.0405472 |
| mature-tRNA-Glu-CTC_5_end | TCCCTGGTGGTCTAGTGCTTAGGATTCGGCGC | 0.0220283 |
| mature-tRNA-Glu-CTC_5_end | TCCCTGGTGGTCAAGTGGTTAGGATTCGG | 1.52E-05 |
| mature-tRNA-Glu-CTC_5_end | TCCCTGGTGGTCTAGTGCTTAGGATTCGG | 0 |
| mature-tRNA-Glu-CTC_5_end | TCCCTGGTGGTCTAGTGTTTAGGATTCGGCG | 3.81E-11 |
| mature-tRNA-Glu-CTC_5_end | TCCCTGGTGGTCTAGTGGTTAGTATTCGG | 6.26E-14 |
| mature-tRNA-Glu-CTC_5_end | TCCCTGGTGGTCTAGTGGTTAGGATTCTG | 2.11E-15 |
| mature-tRNA-Glu-CTC_5_end | TCCCTGCTGGTCTAGTGGTTAGGATTCGGCG | 6.33E-06 |
| mature-tRNA-Glu-CTC_5_end | TCCCTGGTGGTCTAGTGCTTAGGATTCGGC | 0 |
| mature-tRNA-Glu-CTC_5_end | TCCCTGGCGGTCTAGTGGTTAGGATTCGGC | 1.35E-12 |
| mature-tRNA-Glu-CTC_5_end | TCCCTGGAGGTCTAGTGGTTAGGATTCGGCG | 1.94E-07 |
| mature-tRNA-Glu-CTC_5_end | TCCCTGGTGGTCTAGTGTTTAGGATTCGGCGC | 0.0494052 |
| mature-tRNA-Glu-CTC_5_end | TCCCTGGTGGTCTAGTGCTTAGGATTCGGCG | 0 |
| mature-tRNA-Glu-CTC_5_end | TCCCTGGTGGTCGAGTGGTTAGGATTCGG | 0.0459272 |
| mature-tRNA-Glu-CTC_5_end | TCCCTGGTGGTCTAGTCGTTAGGATTCGGCG | 0.0091416 |
| mature-tRNA-Glu-CTC_5_end | TCCCTGGTGGTCTAGTGGTTAGTATTCGGCG | 5.15E-05 |
| mature-tRNA-Glu-CTC_5_end | TCCCTGGTGGTCTAGTGTTTAGGATTCGGCGCT | 0.0091416 |
| mature-tRNA-Glu-CTC_5_end | TCCCTGCTGGTCTAGTGGTTAGGATTCGGC | 0 |
| mature-tRNA-Glu-CTC_5_end | TCCCTGGTGGTCTAGTGGTTAGGATTCCGCG | 7.52E-08 |
| mature-tRNA-Glu-CTC_5_end | TCCCTGCTGGTCTAGTGGTTAGGATTCGG | 0 |
| mature-tRNA-Glu-CTC_5_end | TCCCTGGTGGTCTAGTGCTTAGGATTCGGCGCT | 0.0001429 |
| mature-tRNA-Glu-CTC_5_end | TCCCTGGTGGTCTAGTGGTTACGATTCGG | 4.54E-08 |
| mature-tRNA-Glu-CTC_5_end | TCCCTGGTGGTCTAGTGCTTAGGATTCGGCGCTC | 0.0459272 |
| mature-tRNA-Glu-CTC_5_end | TCCCTGGAGGTCTAGTGGTTAGGATTCGG | 0 |
| mature-tRNA-Glu-CTC_5_end | TCCCTGGTGGTCTAGTGGTTAGGATTCTGC | 2.48E-06 |
| mature-tRNA-Glu-CTC_5_end | TCCCTGGTGGTCTAGTCGTTAGGATTCGG | 8.88E-16 |
| mature-tRNA-Glu-CTC_5_end | TCCCTGGTGGTCTAGTGGTTAGTATTCGGC | 1.60E-14 |
| mature-tRNA-Glu-CTC_5_end | TCCCTGGTGGTCTAGTGATTAGGATTCGG | 0 |
| mature-tRNA-Glu-CTC_5_end | TCCCTGGTGGTCTAGTGATTAGGATTCGGCG | 1.11E-16 |
| mature-tRNA-Glu-CTC_5_end | TCCCTGGTGGTCTAGTGGTTAGGATTCCG | 0 |
| mature-tRNA-Glu-CTC_5_end | TCCCTGGCGGTCTAGTGGTTAGGATTCGGCG | 6.64E-06 |
| mature-tRNA-Glu-CTC_5_end | TCCCTGGTGGTCTAGTGATTAGGATTCGGC | 0 |
| mature-tRNA-Glu-CTC_5_end | TCCCTGGCGGTCTAGTGGTTAGGATTCGG | 0 |
| mature-tRNA-Glu-CTC_5_end | TCCCTGGTGCTCTAGTGGTTAGGATTCGGCG | 1.52E-11 |
| mature-tRNA-Glu-CTC_5_end | TCCCTGGTGGTCTAGTGGTTACGATTCGGC | 6.26E-06 |
| mature-tRNA-Glu-CTC_5_end | TCCCTGGTGGTCTAGTGATTAGGATTCGGCGC | 0.0157469 |
| mature-tRNA-Glu-CTC_5_end | TCCCTGGTGGTCTAGTCGTTAGGATTCGGC | 6.68E-08 |
| mature-tRNA-Glu-CTC_5_end | TCCCTGGAGGTCTAGTGGTTAGGATTCGGCGCT | 0.0087685 |
| mature-tRNA-Glu-CTC_5_end | TCCCTGGTGCTCTAGTGGTTAGGATTCGG | 0 |
| mature-tRNA-Glu-CTC_5_end | TCCCTGGTGCTCTAGTGGTTAGGATTCGGC | 0 |
| mature-tRNA-Glu-CTC_5_end | TCCCTGGTGCTCTAGTGGTTAGGATTCGGCGCT | 0.0054155 |
| mature-tRNA-Glu-CTC_5_end | TCCCTGGCGGTCTAGTGGTTAGGATTCGGCGCT | 0.0463738 |
| mature-tRNA-Glu-CTC_5_end | TCCCTGGTGGTCTAGTGGTTAGGATTCCGC | 0 |
| mature-tRNA-Glu-CTC_5_end | TCCCTGGTGGTCTAGTGGTTAGAATTCGG | 0 |
| mature-tRNA-Glu-CTC_5_end | TCCCTGGGGGTCTAGTGGTTAGGATTCGGC | 0 |
| mature-tRNA-Glu-CTC_5_end | TCCCTGGTGGTCTAGTGGTTAGAATTCGGCG | 4.00E-15 |
| mature-tRNA-Glu-CTC_5_end | TCCCTGGTGGTCTAGTGATTAGGATTCGGCGCT | 0.0001741 |
| mature-tRNA-Glu-CTC_5_end | TCCCTGGGGGTCTAGTGGTTAGGATTCGGCGCT | 0.0280349 |
| mature-tRNA-Glu-CTC_5_end | TCCCTGGGGGTCTAGTGGTTAGGATTCGG | 0 |
| mature-tRNA-Glu-CTC_5_end | TCCCTGGGGGTCTAGTGGTTAGGATTCGGCG | 2.54E-11 |
| mature-tRNA-Glu-CTC_5_end | TCCCTGGTGGTCTAGTGGTTAGAATTCGGC | 0 |
| mature-tRNA-Glu-CTC_5_end | TCCCTGGTGGTCTAGTGGTTAGAATTCGGCGCT | 0.0087685 |
| mature-tRNA-Glu-CTC_5_end | TCCCTGGTGGTCTAGTGGTTAGAATTCGGCGC | 0.012736 |
| mature-tRNA-Glu-TTC_5_end | ACCCATATGGTCTAGCGGTTAGGATTCCTGGTT | 0.0088667 |
| mature-tRNA-Glu-TTC_5_end | ACCCATATGGTCTAGCGGTTAGGATTCCTGGTTT | 0.0086052 |
| mature-tRNA-Glu-TTC_5_end | ACCCACATGGTCTAGCGGTTAGGATTCCTGG | 0.0463513 |
| mature-tRNA-Glu-TTC_5_end | TCCCACATGGTCTAGCGGGTAGGATTCCTG | 0.0276294 |
| mature-tRNA-Glu-TTC_5_end | ACCCATATGGTCTAGCGGTTAGGATTCCTG | 0.0409727 |
| mature-tRNA-Glu-TTC_5_end | TCCCATATGGTCTAGCGGATAGGATTCCTGGTTT | 0.0068572 |
| mature-tRNA-Glu-TTC_5_end | TCCCACATGGTCTAGCGGATAGGATTCCTGGTTT | 0.0253834 |
| mature-tRNA-Glu-TTC_5_end | TCCCACATGGTCTAGCGCTTAGGATTCCTGGTTT | 0.0220283 |
| mature-tRNA-Glu-TTC_5_end | TCCCACATGGTCTAGCGCTTAGGATTCCTGGTT | 0.0459272 |
| mature-tRNA-Glu-TTC_5_end | TCCCATATGGTCTAGCGCTTAGGATTCCTGG | 0.0160965 |
| mature-tRNA-Glu-TTC_5_end | TCCCACATGGTCTAGCGCTTAGGATTCCTGG | 0.0151844 |
| mature-tRNA-Glu-TTC_5_end | TCCCATATGGTCTAGCGCTTAGGATTCCTG | 0.0054155 |
| mature-tRNA-Glu-TTC_5_end | TCCCATATGGTCTAGCGCTTAGGATTCCTGGT | 0.0494052 |
| mature-tRNA-Glu-TTC_5_end | TCCCACATGGTCTAGCGCTTAGGATTCCTG | 0.0054155 |
| mature-tRNA-Glu-TTC_5_end | TCCCATATGGTCTAGCGATTAGGATTCCTG | 0.0243621 |
| mature-tRNA-Glu-TTC_5_end | TCCCATATGCTCTAGCGGTTAGGATTCCTG | 0.0463738 |
| mature-tRNA-Glu-TTC_5_end | TCCCACATGGTCTAGCGGTTAGGATTCCTCGTTT | 0.0005602 |
| mature-tRNA-Glu-TTC_5_end | TCCCATATGGTCTAGCGGTTAGGATTCCTCGTT | 0.0494052 |
| mature-tRNA-Glu-TTC_5_end | TCCCATATGGTCTAGCGGTTAGGATTCCTCGTTT | 0.0005602 |
| mature-tRNA-Glu-TTC_5_end | TCCCACATGGTCTAGCGATTAGGATTCCTGGTTT | 0.0463738 |
| mature-tRNA-Glu-TTC_5_end | TCCCACATGGTCTAGCGATTAGGATTCCTG | 0.0463738 |
| mature-tRNA-Glu-TTC_5_end | TCCCACATGGTCTAGCGATTAGGATTCCTGG | 0.0463738 |
| mature-tRNA-Glu-TTC_5_end | TCCCACATGGTCTAGCGGTTAGGATTCCTCGTT | 0.0034416 |
| mature-tRNA-Glu-TTC_5_end | TCCCACATGGTCTAGCGGTTAGGATTCCTCG | 0.0066316 |
| mature-tRNA-Glu-TTC_5_end | TCCCATATGGTCTAGCGGTTAGGATTCCTCG | 0.0463738 |
| mature-tRNA-Gly-ACC_5_end;mature-tRNA-Val-CAC_5_end;mature-tRNA-Val-AAC_5_end | GTTTCCGTAGTGTAGTGGTTAGCACGTTC | 0.0379437 |
| mature-tRNA-Gly-ACC_5_end;mature-tRNA-Val-CAC_5_end;mature-tRNA-Val-AAC_5_end | GTTTCCGTAGTGTAGTGGTTAGCACGTTCG | 0.0175731 |
| mature-tRNA-Gly-ACC_5_end;mature-tRNA-Val-CAC_5_end;mature-tRNA-Val-AAC_5_end | GTTTCCGTAGTGTAGTGGTTAGCACGTTCGC | 0.013737 |
| mature-tRNA-Gly-CCC_5_end | GCGCCGCTGGTCTAGTGGTATCATGCAAG | 0.0466795 |
| mature-tRNA-Gly-CCC_5_end | GCGCCGCTGGTGTAGTGCTATCATGCAAG | 0.0296818 |
| mature-tRNA-Gly-CCC_5_end | GCGTCGCTGGTGTAGTGGTATCATGCAAG | 0.013737 |
| mature-tRNA-Gly-CCC_5_end | GCGCCGCTGCTGTAGTGGTATCATGCAAGA | 0.0459272 |
| mature-tRNA-Gly-CCC_5_end | GCGCCGCTGCTGTAGTGGTATCATGCAAG | 0.0160965 |
| mature-tRNA-Gly-CCC_5_end | GCGCCGCTGCTGTAGTGGTATCATGCAAGATTC | 0.0280349 |
| mature-tRNA-Gly-CCC_5_end | GCGCCGCTGCTGTAGTGGTATCATGCAAGATT | 0.0494052 |
| mature-tRNA-Gly-CCC_5_end | GCGCCGCTGGTGAAGTGGTATCATGCAAGA | 0.012736 |
| mature-tRNA-Gly-CCC_5_end | GCGCCGCTGGTGCAGTGGTATCATGCAAG | 0.0243621 |
| mature-tRNA-Gly-CCC_5_end | GCGCCGCTGGTGAAGTGGTATCATGCAAGATT | 0.0463738 |
| mature-tRNA-Gly-CCC_5_end | GCGCCGCTGGTGAAGTGGTATCATGCAAG | 0.001781 |
| mature-tRNA-Gly-CCC_5_end | GCGCCGCTGGTGAAGTGGTATCATGCAAGATTC | 0.001781 |
| mature-tRNA-Gly-CCC_5_end | GCGCCGCTGGTGGAGTGGTATCATGCAAGA | 0.0066316 |
| mature-tRNA-Gly-CCC_5_end | GCGCCGCTGGTGGAGTGGTATCATGCAAGATT | 0.0243621 |
| mature-tRNA-Gly-CCC_5_end | GCGCCGCTGGTGGAGTGGTATCATGCAAGATTC | 0.0066316 |
| mature-tRNA-Gly-CCC_5_end | GCGCCGCTGGTGGAGTGGTATCATGCAAG | 0.001781 |
| mature-tRNA-Gly-GCC_5_end | GCATTGGTGGTGCAGTGGTAGAATTCTCGC | 1.92E-13 |
| mature-tRNA-Gly-GCC_5_end | GCATTGGTGGTACAGTGGTAGAATTCTCGCC | 4.48E-11 |
| mature-tRNA-Gly-GCC_5_end | GCATTGGTGGTACAGTGGTAGAATTCTCGC | 8.10E-15 |
| mature-tRNA-Gly-GCC_5_end | GCATTGGTGGTGCAGTGGTAGAATTCTCG | 0 |
| mature-tRNA-Gly-GCC_5_end | GCATTGGTGGTGCAGTGGTAGAATTCTCGCC | 5.21E-09 |
| mature-tRNA-Gly-GCC_5_end | GCATTGGTGGTACAGTGGTAGAATTCTCG | 0 |
| mature-tRNA-Gly-GCC_5_end | GCATTGGTGGTCCAGTGGTAGAATTCTCGC | 6.07E-11 |
| mature-tRNA-Gly-GCC_5_end | GCATTGGTGGTCCAGTGGTAGAATTCTCGCC | 5.99E-08 |
| mature-tRNA-Gly-GCC_5_end | GCATTGGTGGTCCAGTGGTAGAATTCTCG | 0 |
| mature-tRNA-Gly-GCC_5_end | GCAATGGTGGTTCAGTGGTAGAATTCTCGC | 0.0066972 |
| mature-tRNA-Gly-GCC_5_end | GCATTGGTGGTTCAGTGGTTGAATTCTCGC | 0.0245493 |
| mature-tRNA-Gly-GCC_5_end | GCAATGGTGGTTCAGTGGTAGAATTCTCGCC | 0.0245493 |
| mature-tRNA-Gly-GCC_5_end | GCAATGGTGGTTCAGTGGTAGAATTCTCG | 0.0001776 |
| mature-tRNA-Gly-GCC_5_end | GCATTGGTGGTTCAGTGGTTGAATTCTCG | 0.0054889 |
| mature-tRNA-Gly-GCC_5_end | GCATTGGTTGTTCAGTGGTAGAATTCTCG | 2.59E-10 |
| mature-tRNA-Gly-GCC_5_end | GCATTGGTTGTTCAGTGGTAGAATTCTCGC | 2.65E-05 |
| mature-tRNA-Gly-GCC_5_end | GCATTGGTTGTTCAGTGGTAGAATTCTCGCC | 0.0002146 |
| mature-tRNA-Gly-GCC_5_end | GCATTGGTCGTTCAGTGGTAGAATTCTCGCC | 0.0005594 |
| mature-tRNA-Gly-GCC_5_end | GCATTGGTCGTTCAGTGGTAGAATTCTCG | 1.49E-09 |
| mature-tRNA-Gly-GCC_5_end | GCATTGGTCGTTCAGTGGTAGAATTCTCGC | 4.36E-05 |
| mature-tRNA-Gly-GCC_5_end | GCAGTGGTGGTTCAGTGGTAGAATTCTCG | 0.0165753 |
| mature-tRNA-Gly-GCC_5_end | GCATTGGTGGTTCAGTGGAAGAATTCTCGC | 0.0124678 |
| mature-tRNA-Gly-GCC_5_end | GCATTGGTGATTCAGTGGTAGAATTCTCG | 4.18E-10 |
| mature-tRNA-Gly-GCC_5_end | TCATTGGTGGTTCAGTGGTAGAATTCTCG | 0.0264906 |
| mature-tRNA-Gly-GCC_5_end | GCATTGATGGTTCAGTGGTAGAATTCTCGC | 0.0125301 |
| mature-tRNA-Gly-GCC_5_end | GCATTGATGGTTCAGTGGTAGAATTCTCG | 0.0010481 |
| mature-tRNA-Gly-GCC_5_end | GCATTGCTGGTTCAGTGGTAGAATTCTCGCC | 0.0259311 |
| mature-tRNA-Gly-GCC_5_end | GCATTGGTGGTTCAGTGTTAGAATTCTCGCC | 0.003872 |
| mature-tRNA-Gly-GCC_5_end | GCATTGCTGGTTCAGTGGTAGAATTCTCG | 3.30E-05 |
| mature-tRNA-Gly-GCC_5_end | GCATTGGTGGTTCAGTGCTAGAATTCTCGC | 1.09E-06 |
| mature-tRNA-Gly-GCC_5_end | GCATTCGTGGTTCAGTGGTAGAATTCTCGC | 0.0011422 |
| mature-tRNA-Gly-GCC_5_end | GCATTGGTGGTTCAGTGTTAGAATTCTCG | 8.87E-09 |
| mature-tRNA-Gly-GCC_5_end | GCATTCGTGGTTCAGTGGTAGAATTCTCGCC | 0.0033339 |
| mature-tRNA-Gly-GCC_5_end | GCATTGGTGCTTCAGTGGTAGAATTCTCG | 0 |
| mature-tRNA-Gly-GCC_5_end | GCATTCGTGGTTCAGTGGTAGAATTCTCG | 4.29E-08 |
| mature-tRNA-Gly-GCC_5_end | GCATTGGTGGTTCAGTGCTAGAATTCTCG | 0 |
| mature-tRNA-Gly-GCC_5_end | GCATTGGCGGTTCAGTGGTAGAATTCTCGC | 0.0146733 |
| mature-tRNA-Gly-GCC_5_end | GCATTGGTGCTTCAGTGGTAGAATTCTCGC | 1.00E-07 |
| mature-tRNA-Gly-GCC_5_end | GCATTGGCGGTTCAGTGGTAGAATTCTCGCC | 0.0456404 |
| mature-tRNA-Gly-GCC_5_end | GCATTGGTGGTTCAGTGGTAGAATTCTCC | 4.67E-09 |
| mature-tRNA-Gly-GCC_5_end | GCATTGGTGGTTCAGTGCTAGAATTCTCGCC | 1.00E-07 |
| mature-tRNA-Gly-GCC_5_end | GCATTGGAGGTTCAGTGGTAGAATTCTCGCC | 0.0050736 |
| mature-tRNA-Gly-GCC_5_end | GCATTGGTGGTTCAGTGGTAGAATTCTCT | 0.0105406 |
| mature-tRNA-Gly-GCC_5_end | GCATTGGAGGTTCAGTGGTAGAATTCTCGC | 0.0001953 |
| mature-tRNA-Gly-GCC_5_end | GCATTGGTGGTTCAGTGGTAGAATTCTCCC | 0.0001119 |
| mature-tRNA-Gly-GCC_5_end | GCATTGGTGGTTCAGTGGTAGAATTCTCCCC | 0.0042716 |
| mature-tRNA-Gly-GCC_5_end | GCATTGGTGGTTCAGTGATAGAATTCTCGC | 2.52E-07 |
| mature-tRNA-Gly-GCC_5_end | GCATTGGTGGTTCAGTCGTAGAATTCTCG | 0.0220283 |
| mature-tRNA-Gly-GCC_5_end | GCATTGGTGCTTCAGTGGTAGAATTCTCGCC | 1.28E-07 |
| mature-tRNA-Gly-GCC_5_end | GCATTGGAGGTTCAGTGGTAGAATTCTCG | 1.61E-11 |
| mature-tRNA-Gly-GCC_5_end | GCATTGGTGGTTCAGTGCTAGAATTCTCGCCT | 0.0405472 |
| mature-tRNA-Gly-GCC_5_end | GCATTGGTGGTTCAGTGATAGAATTCTCG | 0 |
| mature-tRNA-Gly-GCC_5_end | GCATGGGGGGTTCAGTGGTAGAATTCTCG | 0.0160965 |
| mature-tRNA-Gly-GCC_5_end | GCATTGGTGGTTCAGTGATAGAATTCTCGCC | 6.85E-08 |
| mature-tRNA-Gly-GCC_5_end | GCATTGGGGGTTCAGTGGTAGAATTCTCGCC | 0.000665 |
| mature-tRNA-Gly-GCC_5_end | GCATTGGCGGTTCAGTGGTAGAATTCTCG | 1.64E-08 |
| mature-tRNA-Gly-GCC_5_end | GCATTGGTGCTTCAGTGGTAGAATTCTCGCCT | 0.0494052 |
| mature-tRNA-Gly-GCC_5_end | GCATTGGGGGTTCAGTGGTAGAATTCTCGC | 1.11E-05 |
| mature-tRNA-Gly-GCC_5_end | GCATTGGGGGTTCAGTGGTAGAATTCTCG | 6.44E-15 |
| mature-tRNA-Gly-GCC_5_end | GCATTGGTGGTTCAGTGATAGAATTCTCGCCT | 0.0494052 |
| mature-tRNA-Gly-GCC_5_end | GCATTGGTGGTTCTGTGGTAGAATTCTCG | 6.07E-11 |
| mature-tRNA-Gly-GCC_5_end | GCATTGGTGGTTCTGTGGTAGAATTCTCGC | 4.75E-06 |
| mature-tRNA-Gly-GCC_5_end | GCATTGGTGGTTCTGTGGTAGAATTCTCGCC | 0.0007893 |
| mature-tRNA-Gly-GCC_5_end;mature-tRNA-Gly-CCC_5_end | GCATTGGTAGTTCAGTGGTAGAATTCTCGCC | 0.0104007 |
| mature-tRNA-Gly-GCC_5_end;mature-tRNA-Gly-CCC_5_end | GCATTGGTAGTTCAGTGGTAGAATTCTCGC | 0.003493 |
| mature-tRNA-Gly-TCC_5_end | GCGTTGGTGGTCTAGTGGTGAGCATAGCTG | 0.0466795 |
| mature-tRNA-Gly-TCC_5_end | GCGTTGGTGGTGTAGTGGTGAGCATAGCTG | 0.0466795 |
| mature-tRNA-Gly-TCC_5_end | GCGTTGGTGGTATAGTGCTGAGCATAGCTG | 0.0456404 |
| mature-tRNA-Gly-TCC_5_end | GCGTTCGTGGTATAGTGGTGAGCATAGCTG | 0.0349247 |
| mature-tRNA-Gly-TCC_5_end | GCGTTGGTGCTATAGTGGTGAGCATAGCT | 0.0494052 |
| mature-tRNA-Gly-TCC_5_end | GCGTTGGTGCTATAGTGGTGAGCATAGCTG | 0.0160965 |
| mature-tRNA-His-GTG_5_end | GGCCGTGATCGGATAGTGGTTAGTACTCTGC | 0.0466795 |
| mature-tRNA-His-GTG_5_end | GGCCGTGATCGCATAGTGGTTAGTACTCTGC | 0.0466795 |
| mature-tRNA-His-GTG_5_end | GGCCGTGATCGAATAGTGGTTAGTACTCTGC | 0.0245493 |
| mature-tRNA-His-GTG_5_end | GGTCGTGATCGTATAGTGGTTAGTACTCTGC | 0.0024578 |
| mature-tRNA-His-GTG_5_end | GGTCGTGATCGTATAGTGGTTAGTACTCTGCGTT | 0.0456404 |
| mature-tRNA-His-GTG_5_end | GGTCGTGATCGTATAGTGGTTAGTACTCTGCG | 0.0067401 |
| mature-tRNA-His-GTG_5_end | GGCCGTGATCGTATAGCGGTTAGTACTCTGC | 0.0463738 |
| mature-tRNA-His-GTG_5_end | GGCCGTGATCGTATAGTGGTTAGTACTCTCC | 0.0463738 |
| mature-tRNA-iMet-CAT_5_end | AGCAGAGTGGCTCAGCGGAAGCGTGCTGGG | 0.0009316 |
| mature-tRNA-iMet-CAT_5_end | AGCAGAGTGGCCCAGCGGAAGCGTGCTGGGC | 0.0466795 |
| mature-tRNA-iMet-CAT_5_end | AGCAGAGTGGCCCAGCGGAAGCGTGCTGGG | 0.0008016 |
| mature-tRNA-iMet-CAT_5_end | AGCAGAGTGGCACAGCGGAAGCGTGCTGG | 0.0128479 |
| mature-tRNA-iMet-CAT_5_end | AGCAGAGTGGCACAGCGGAAGCGTGCTGGG | 0.0003745 |
| mature-tRNA-iMet-CAT_5_end | AGCAGAGTGGCGCAGCGTAAGCGTGCTGGG | 0.0176164 |
| mature-tRNA-iMet-CAT_5_end | AGCAGAGTGGCGCAGCGCAAGCGTGCTGG | 0.0315061 |
| mature-tRNA-iMet-CAT_5_end | AGCAGAGTGGCGCAGCGCAAGCGTGCTGGG | 5.65E-05 |
| mature-tRNA-iMet-CAT_5_end | AGCAGAGTGGCGCAGCGGAAGCGTGCTGGC | 0.0015287 |
| mature-tRNA-iMet-CAT_5_end | AGCACAGTGGCGCAGCGGAAGCGTGCTGGG | 0.0456404 |
| mature-tRNA-iMet-CAT_5_end | AGCAGAGTGGCGCAGCGGAAGCGTGCTGCG | 0.0001659 |
| mature-tRNA-iMet-CAT_5_end | AGCAGAGTGGCGCAGCGCAAGCGTGCTGGGC | 0.000772 |
| mature-tRNA-iMet-CAT_5_end | AGCAGAGTGGCGCAGCGGAAGCGTGCTGTG | 0.0121267 |
| mature-tRNA-iMet-CAT_5_end | AGCAGAGTGGCGCAGCGTAAGCGTGCTGGGC | 0.0220283 |
| mature-tRNA-iMet-CAT_5_end | AGCAGAGTGGCGCAGCGAAAGCGTGCTGGG | 0.0002512 |
| mature-tRNA-iMet-CAT_5_end | AGCAGAGTGGCGCAGCGAAAGCGTGCTGGGC | 0.013737 |
| mature-tRNA-iMet-CAT_5_end | AGCAGAGTGGCGCAGCGGAAGCGTGCTGGCC | 0.0042716 |
| mature-tRNA-iMet-CAT_5_end | AGCAGAGTGGCGCTGCGGAAGCGTGCTGGG | 0.0029431 |
| mature-tRNA-iMet-CAT_5_end | AGCAGAGAGGCGCAGCGGAAGCGTGCTGGG | 0.0084785 |
| mature-tRNA-iMet-CAT_5_end | AGCAGAGTGGCGCAGCGGAAGCGTGCTGCGC | 0.0091416 |
| mature-tRNA-iMet-CAT_5_end | AGCAGAGGGGCGCAGCGGAAGCGTGCTGGG | 0.0160965 |
| mature-tRNA-iMet-CAT_5_end | AGCAGAGCGGCGCAGCGGAAGCGTGCTGGG | 0.0157469 |
| mature-tRNA-iMet-CAT_5_end | AGCAGAGTGGCGCTGCGGAAGCGTGCTGGGC | 0.0280349 |
| mature-tRNA-Leu-CAG_5_end | GTCAGGATGGCAGAGCGGTCTAAGGCGCTGCGTT | 0.0066972 |
| mature-tRNA-Leu-CAG_5_end | GTCAGGTTGGCCGAGCGGTCTAAGGCGCTGCGTT | 0.0066972 |
| mature-tRNA-Leu-CAG_5_end | GTCAGGATGGCCGAGCGGGCTAAGGCGCTGCGTT | 0.0162798 |
| mature-tRNA-Leu-CAG_5_end | GTCAGGATGGCCGAGCGGACTAAGGCGCTGCGTT | 0.0004087 |
| mature-tRNA-Leu-CAG_5_end | GTCAGGATGGCCGAGCGGTCTAAGGCGCTCCGTT | 0.0157469 |
| mature-tRNA-Lys-CTT_5_end | GCCCGGCTAGCTCAGTCCGTAGAGCATGAGAC | 0.0459272 |
| mature-tRNA-Lys-CTT_5_end | GCCCGGCTAGCTCAGTCCGTAGAGCATGAGA | 0.0280349 |
| mature-tRNA-Val-AAC_5_end | GTTTCCGTAGTATAGTGGTTATCACATTC | 0.0034794 |
| mature-tRNA-Val-AAC_5_end | GTTTCCGTAGTTTAGTGGTTATCACATTCG | 0.0466795 |
| mature-tRNA-Val-AAC_5_end | GTTTCCGTAGTATAGTGGTTATCACATTCGCC | 0.0466795 |
| mature-tRNA-Val-AAC_5_end | GTTTCCGTAGTATAGTGGTTATCACATTCGC | 0.0018026 |
| mature-tRNA-Val-AAC_5_end | GTTTCCGTAGTTTAGTGGTTATCACATTCGC | 0.0245493 |
| mature-tRNA-Val-AAC_5_end | GTTTCCGTAGTTTAGTGGTTATCACATTC | 0.0466795 |
| mature-tRNA-Val-AAC_5_end | GTTTCCGTAGTATAGTGGTTATCACATTCG | 0.0034794 |
| mature-tRNA-Val-AAC_5_end | GTTTCCGTAGTATAGTGGTTATCACATTCGCCT | 0.0466795 |
| mature-tRNA-Val-AAC_5_end | GTTTCCGTAGTCTAGTGGTTATCACATTCG | 0.0034794 |
| mature-tRNA-Val-AAC_5_end | GTTTCCGTAGTCTAGTGGTTATCACATTC | 0.0066972 |
| mature-tRNA-Val-AAC_5_end | GTTTCCGTAGTCTAGTGGTTATCACATTCGC | 0.0066972 |
| mature-tRNA-Val-AAC_5_end | GTTTCCGTCGTGTAGTGGTTATCACATTC | 0.0466795 |
| mature-tRNA-Val-AAC_5_end | GTTTCCGTCGTGTAGTGGTTATCACATTCGC | 0.0245493 |
| mature-tRNA-Val-AAC_5_end | GTTTCCGTCGTGTAGTGGTTATCACATTCG | 0.0245493 |
| mature-tRNA-Val-AAC_5_end | GTTTCCGTGGTGTAGTGGTTATCACATTCGC | 0.0466795 |
| mature-tRNA-Val-AAC_5_end | GTTTCCGTGGTGTAGTGGTTATCACATTCG | 0.0466795 |
| mature-tRNA-Val-AAC_5_end | GTTTCCGTAGTGTAGTGCTTATCACATTCG | 0.0492036 |
| mature-tRNA-Val-AAC_5_end | GTTTCCGTAGTGTAGTGATTATCACATTCG | 0.0296818 |
| mature-tRNA-Val-AAC_5_end | GTTTCCGTAGTGTAGTGCTTATCACATTCGCCT | 0.0067401 |
| mature-tRNA-Val-AAC_5_end | GTTTCCGTAGTGTAGTGATTATCACATTCGC | 0.0296818 |
| mature-tRNA-Val-AAC_5_end | GTTTCCGCAGTGTAGTGGTTATCACATTC | 0.0405472 |
| mature-tRNA-Val-AAC_5_end | GTTTCCGTAGTGTAGTGCTTATCACATTCGC | 0.0013598 |
| mature-tRNA-Val-AAC_5_end | GTTTCCGTAGTGTAGTGCTTATCACATTCGCC | 0.0076325 |
| mature-tRNA-Val-AAC_5_end | GTTTCCGTAGTGTAGTGATTATCACATTCGCC | 0.0273475 |
| mature-tRNA-Val-AAC_5_end | GTTTCCGTAGTGTAGTGGTTATCACATTCGCGT | 0.0273475 |
| mature-tRNA-Val-AAC_5_end | GTTTCCGTAGTGTAGTGGTTATCACATTCGCA | 0.0051838 |
| mature-tRNA-Val-AAC_5_end | GTTTCCGTAGTGTAGTGGTTATCACATTCC | 0.0002366 |
| mature-tRNA-Val-AAC_5_end | GTTTCCGTAGTGTAGTGATTATCACATTCGCCT | 0.0093785 |
| mature-tRNA-Val-AAC_5_end | GTTTCCGCAGTGTAGTGGTTATCACATTCG | 0.0093785 |
| mature-tRNA-Val-AAC_5_end | GTTTCCGAAGTGTAGTGGTTATCACATTCG | 0.001128 |
| mature-tRNA-Val-AAC_5_end | GTTTCCGAAGTGTAGTGGTTATCACATTC | 0.0018916 |
| mature-tRNA-Val-AAC_5_end | GTTTCCGTAGTGTAGTGGTTATCACATTCGCG | 0.0494052 |
| mature-tRNA-Val-AAC_5_end | GTTTCCGAAGTGTAGTGGTTATCACATTCGCC | 0.0032239 |
| mature-tRNA-Val-AAC_5_end | GTTTCCGCAGTGTAGTGGTTATCACATTCGCCT | 0.0031022 |
| mature-tRNA-Val-AAC_5_end | GTTTCCGTAGTGTAGTGGTTATCACATTCCCCT | 9.21E-08 |
| mature-tRNA-Val-AAC_5_end | GTTTCCGTAGTGTAGTGGTTATCACATTCCC | 1.19E-08 |
| mature-tRNA-Val-AAC_5_end | GTTTCCGCAGTGTAGTGGTTATCACATTCGCC | 0.0048463 |
| mature-tRNA-Val-AAC_5_end | GTTTCCGTAGTGTAGTGGTTATCACATTCGCAT | 0.0009971 |
| mature-tRNA-Val-AAC_5_end | GTTTCCGCAGTGTAGTGGTTATCACATTCGC | 0.0001741 |
| mature-tRNA-Val-AAC_5_end | GTTTCCGGAGTGTAGTGGTTATCACATTC | 0.0054155 |
| mature-tRNA-Val-AAC_5_end | GTTTCCGAAGTGTAGTGGTTATCACATTCGC | 8.56E-07 |
| mature-tRNA-Val-AAC_5_end | GTTTCCGTAGTGTAGTGGTTATCACATTCGCTT | 0.0007893 |
| mature-tRNA-Val-AAC_5_end | GTTTCCGTAGTGTAGTGGTTATCACATTCCCC | 2.15E-05 |
| mature-tRNA-Val-AAC_5_end | GTTTCCGGAGTGTAGTGGTTATCACATTCG | 0.0004269 |
| mature-tRNA-Val-AAC_5_end | GTTTCCGAAGTGTAGTGGTTATCACATTCGCCT | 2.92E-05 |
| mature-tRNA-Val-AAC_5_end | GTTTCCGTAGTGCAGTGGTTATCACATTC | 0.0026612 |
| mature-tRNA-Val-AAC_5_end | GTTTCCGGAGTGTAGTGGTTATCACATTCGCC | 0.0007893 |
| mature-tRNA-Val-AAC_5_end | GTTTCCGTAGTGCAGTGGTTATCACATTCG | 0.0004269 |
| mature-tRNA-Val-AAC_5_end | GTTTCCGGAGTGTAGTGGTTATCACATTCGCCT | 0.0004269 |
| mature-tRNA-Val-AAC_5_end | GTTTCCGTAGTGAAGTGGTTATCACATTCG | 0.0026612 |
| mature-tRNA-Val-AAC_5_end | GTTTCCGTAGTGCAGTGGTTATCACATTCGCC | 0.0243621 |
| mature-tRNA-Val-AAC_5_end | GTTTCCGTAGTGAAGTGGTTATCACATTC | 0.0009195 |
| mature-tRNA-Val-AAC_5_end | GTTTCCGGAGTGTAGTGGTTATCACATTCGC | 5.25E-06 |
| mature-tRNA-Val-AAC_5_end | GTTTCCGTAGTGAAGTGGTTATCACATTCGCCT | 0.0004737 |
| mature-tRNA-Val-AAC_5_end | GTTTCCGTAGTGCAGTGGTTATCACATTCGC | 0.0009195 |
| mature-tRNA-Val-AAC_5_end | GTTTCCGTAGTGGAGTGGTTATCACATTC | 0.0034416 |
| mature-tRNA-Val-AAC_5_end | GTTTCCGTAGTGGAGTGGTTATCACATTCGCC | 0.012736 |
| mature-tRNA-Val-AAC_5_end | GTTTCCGTAGTGCAGTGGTTATCACATTCGCCT | 0.0243621 |
| mature-tRNA-Val-AAC_5_end | GTTTCCGTAGTGAAGTGGTTATCACATTCGC | 3.28E-05 |
| mature-tRNA-Val-AAC_5_end | GTTTCCGTAGTGGAGTGGTTATCACATTCG | 0.0034416 |
| mature-tRNA-Val-AAC_5_end | GTTTCCGTAGTGAAGTGGTTATCACATTCGCC | 0.001781 |
| mature-tRNA-Val-AAC_5_end | GTTTCCGTAGTGGAGTGGTTATCACATTCGCCT | 0.012736 |
| mature-tRNA-Val-AAC_5_end | GTTTCCGTAGTGGAGTGGTTATCACATTCGC | 0.0002436 |
| mature-tRNA-Val-AAC_5_end;mature-tRNA-Val-CAC_5_end | GTTTCCGTAGTTTAGTGGTTATCACGTTCG | 0 |
| mature-tRNA-Val-AAC_5_end;mature-tRNA-Val-CAC_5_end | GTTTCCGTAGTATAGTGGTTATCACGTTCGC | 0 |
| mature-tRNA-Val-AAC_5_end;mature-tRNA-Val-CAC_5_end | GTTTCCGTAGTTTAGTGGTTATCACGTTCGC | 0 |
| mature-tRNA-Val-AAC_5_end;mature-tRNA-Val-CAC_5_end | GTTTCCGTAGTCTAGTGGTTATCACGTTCGCCT | 0 |
| mature-tRNA-Val-AAC_5_end;mature-tRNA-Val-CAC_5_end | GTTTCCGTCGTGTAGTGGTTATCACGTTCGCC | 0 |
| mature-tRNA-Val-AAC_5_end;mature-tRNA-Val-CAC_5_end | GTTTCCGTCGTGTAGTGGTTATCACGTTCGC | 0 |
| mature-tRNA-Val-AAC_5_end;mature-tRNA-Val-CAC_5_end | GTTTCCGTAGTGTAGTGGTTATGACGTTC | 0 |
| mature-tRNA-Val-AAC_5_end;mature-tRNA-Val-CAC_5_end | GTTTCCGTGGTGTAGTGGTTATCACGTTCGCC | 0 |
| mature-tRNA-Val-AAC_5_end;mature-tRNA-Val-CAC_5_end | GTTACCGTAGTGTAGTGGTTATCACGTTC | 2.46E-13 |
| mature-tRNA-Val-AAC_5_end;mature-tRNA-Val-CAC_5_end | GTTACCGTAGTGTAGTGGTTATCACGTTCG | 0 |
| mature-tRNA-Val-AAC_5_end;mature-tRNA-Val-CAC_5_end | GATTCCGTAGTGTAGTGGTTATCACGTTCGCC | 8.66E-15 |
| mature-tRNA-Val-AAC_5_end;mature-tRNA-Val-CAC_5_end | GTTGCCGTAGTGTAGTGGTTATCACGTTCG | 2.45E-14 |
| mature-tRNA-Val-AAC_5_end;mature-tRNA-Val-CAC_5_end | GTTTCCGTAGTGTAGTGGTTTTCACGTTC | 0.0001454 |
| mature-tRNA-Val-AAC_5_end;mature-tRNA-Val-CAC_5_end | GATTCCGTAGTGTAGTGGTTATCACGTTCGC | 0 |
| mature-tRNA-Val-AAC_5_end;mature-tRNA-Val-CAC_5_end | GTTTCCGTAGTGTAGTGGTTATCTCGTTCGCC | 2.21E-13 |
| mature-tRNA-Val-AAC_5_end;mature-tRNA-Val-CAC_5_end | GTTGCCGTAGTGTAGTGGTTATCACGTTCGCC | 5.17E-05 |
| mature-tRNA-Val-AAC_5_end;mature-tRNA-Val-CAC_5_end | GTTGCCGTAGTGTAGTGGTTATCACGTTCGC | 1.60E-08 |
| mature-tRNA-Val-AAC_5_end;mature-tRNA-Val-CAC_5_end | GTTTCCGTAGTGTAGTGGGTATCACGTTC | 0 |
| mature-tRNA-Val-AAC_5_end;mature-tRNA-Val-CAC_5_end | GTTTCCGTAGTGTAGTGGTTATAACGTTCG | 2.38E-07 |
| mature-tRNA-Val-AAC_5_end;mature-tRNA-Val-CAC_5_end | GTTTCCGTAGTGTAGTGGATATCACGTTCGC | 0 |
| mature-tRNA-Val-AAC_5_end;mature-tRNA-Val-CAC_5_end | GTTTCCGTAGTGTAGTGGTTATAACGTTCGCC | 0.0219684 |
| mature-tRNA-Val-AAC_5_end;mature-tRNA-Val-CAC_5_end | GTGTCCGTAGTGTAGTGGTTATCACGTTCG | 1.53E-05 |
| mature-tRNA-Val-AAC_5_end;mature-tRNA-Val-CAC_5_end | GTTCCCGTAGTGTAGTGGTTATCACGTTC | 8.41E-05 |
| mature-tRNA-Val-AAC_5_end;mature-tRNA-Val-CAC_5_end | TTTTCCGTAGTGTAGTGGTTATCACGTTCG | 5.81E-08 |
| mature-tRNA-Val-AAC_5_end;mature-tRNA-Val-CAC_5_end | GTTTCCGTACTGTAGTGGTTATCACGTTC | 1.29E-10 |
| mature-tRNA-Val-AAC_5_end;mature-tRNA-Val-CAC_5_end | GTTTCCGTAGTGTAGTGTTTATCACGTTCGC | 0 |
| mature-tRNA-Val-AAC_5_end;mature-tRNA-Val-CAC_5_end | GTTTCCGTATTGTAGTGGTTATCACGTTCGCC | 0.0178969 |
| mature-tRNA-Val-AAC_5_end;mature-tRNA-Val-CAC_5_end | GTTTACGTAGTGTAGTGGTTATCACGTTCG | 3.39E-08 |
| mature-tRNA-Val-AAC_5_end;mature-tRNA-Val-CAC_5_end | GTTTCCGTAGTGTAGTGTTTATCACGTTCGCCT | 0 |
| mature-tRNA-Val-AAC_5_end;mature-tRNA-Val-CAC_5_end | GTTTACGTAGTGTAGTGGTTATCACGTTC | 1.21E-06 |
| mature-tRNA-Val-AAC_5_end;mature-tRNA-Val-CAC_5_end | GTTTCCGTAGTGTAGTGCTTATCACGTTCGC | 0 |
| mature-tRNA-Val-AAC_5_end;mature-tRNA-Val-CAC_5_end | GTTTCCGTAGTGTAGTGGTTATCACGTTCT | 0 |
| mature-tRNA-Val-AAC_5_end;mature-tRNA-Val-CAC_5_end | GTTTCCGTAGTGTAGTGGTTATCAGGTTCG | 0.0005989 |
| mature-tRNA-Val-AAC_5_end;mature-tRNA-Val-CAC_5_end | GTTTCCGTAGTGTAGTGCTTATCACGTTCGCCT | 0 |
| mature-tRNA-Val-AAC_5_end;mature-tRNA-Val-CAC_5_end | GTTTCCGTAGTGTAGTGGTTATCACGTTCGCGT | 0 |
| mature-tRNA-Val-AAC_5_end;mature-tRNA-Val-CAC_5_end | GTTTCCGTAGTGTAGTGGTTATCAAGTTCGC | 0.0076325 |
| mature-tRNA-Val-AAC_5_end;mature-tRNA-Val-CAC_5_end | GTTTCCGTAGTGTAGTGATTATCACGTTCGCC | 0 |
| mature-tRNA-Val-AAC_5_end;mature-tRNA-Val-CAC_5_end | GTTTCCGTACTGTAGTGGTTATCACGTTCGCCT | 0 |
| mature-tRNA-Val-AAC_5_end;mature-tRNA-Val-CAC_5_end | GTTTACGTAGTGTAGTGGTTATCACGTTCGCCT | 5.56E-06 |
| mature-tRNA-Val-AAC_5_end;mature-tRNA-Val-CAC_5_end | GTTTCCGTAGTGTAGTGGTTATCAGGTTCGCC | 0.0249587 |
| mature-tRNA-Val-AAC_5_end;mature-tRNA-Val-CAC_5_end | GTTTCCGTAGTGTAGTGGTTATCAAGTTCGCCT | 0.0459272 |
| mature-tRNA-Val-AAC_5_end;mature-tRNA-Val-CAC_5_end | GTTTCCGTAGTGTAGTGGTCATCACGCTCCCC | 0.0459272 |
| mature-tRNA-Val-AAC_5_end;mature-tRNA-Val-CAC_5_end | GTTTCCGTAGTGTAGTGCTCATCACGCTCGCC | 0.0280349 |
| mature-tRNA-Val-AAC_5_end;mature-tRNA-Val-CAC_5_end | GTTTCCGTAGTGTAGTGGTTAACACGTTCG | 0 |
| mature-tRNA-Val-AAC_5_end;mature-tRNA-Val-CAC_5_end | GTTTCCGAAGTGTAGTGGTCATCACGCTCGC | 0.0463738 |
| mature-tRNA-Val-AAC_5_end;mature-tRNA-Val-CAC_5_end | GTTTCCGAAGTGTAGTGGTTATCACGTTC | 0 |
| mature-tRNA-Val-AAC_5_end;mature-tRNA-Val-CAC_5_end | GTTTCCGAAGTGTAGTGGTTATCACGTTCG | 0 |
| mature-tRNA-Val-AAC_5_end;mature-tRNA-Val-CAC_5_end | GTTTCCGAAGTGTAGTGGTTATCACGTTCGCC | 0 |
| mature-tRNA-Val-AAC_5_end;mature-tRNA-Val-CAC_5_end | GTTTCCGTAGTGTAGTGATCATCACGCTCGCC | 0.0463738 |
| mature-tRNA-Val-AAC_5_end;mature-tRNA-Val-CAC_5_end | GTTTCCGCAGTGTAGTGGTTATCACGTTCGCCT | 0 |
| mature-tRNA-Val-AAC_5_end;mature-tRNA-Val-CAC_5_end | GTTTCCGAAGTGTAGTGGTCATCACGCTCGCCT | 0.012736 |
| mature-tRNA-Val-AAC_5_end;mature-tRNA-Val-CAC_5_end | GTTTCCGAAGTGTAGTGGTTATCACGTTCGCCT | 0 |
| mature-tRNA-Val-AAC_5_end;mature-tRNA-Val-CAC_5_end | GTTTCCGTAGTGCAGTGGTTATCACGTTCG | 0 |
| mature-tRNA-Val-AAC_5_end;mature-tRNA-Val-CAC_5_end | GTTTCCGTAGTGTAGTGGTTATCACGTTCGCAT | 0 |
| mature-tRNA-Val-AAC_5_end;mature-tRNA-Val-CAC_5_end | GTTTCCGGAGTGTAGTGGTTATCACGTTCGCC | 0 |
| mature-tRNA-Val-AAC_5_end;mature-tRNA-Val-CAC_5_end | GTTTGCGTAGTGTAGTGGTTATCACGTTC | 0 |
| mature-tRNA-Val-AAC_5_end;mature-tRNA-Val-CAC_5_end | GTTTGCGTAGTGTAGTGGTTATCACGTTCG | 0 |
| mature-tRNA-Val-AAC_5_end;mature-tRNA-Val-CAC_5_end | GTTTCCGCAGTGTAGTGGTCATCACGCTCGCCT | 0.0463738 |
| mature-tRNA-Val-AAC_5_end;mature-tRNA-Val-CAC_5_end | GTTTGCGTAGTGTAGTGGTTATCACGTTCGC | 0 |
| mature-tRNA-Val-AAC_5_end;mature-tRNA-Val-CAC_5_end | GTTTGCGTAGTGTAGTGGTTATCACGTTCGCC | 0 |
| mature-tRNA-Val-AAC_5_end;mature-tRNA-Val-CAC_5_end | GTTTCCGGAGTGTAGTGGTCATCACGCTCGCC | 0.0463738 |
| mature-tRNA-Val-AAC_5_end;mature-tRNA-Val-CAC_5_end | GTTTCCGTAGTGGAGTGGTTATCACGTTCGCCT | 0 |
| mature-tRNA-Val-CAC_5_end | GTTTCCGTAGTATAGTGGTTATCACGTTCGCCTC | 3.02E-07 |
| mature-tRNA-Val-CAC_5_end | GTTTCCGTAGTTTAGTGGTTATCACGTTCGCCTC | 6.52E-05 |
| mature-tRNA-Val-CAC_5_end | GTTTCCGTAGTCTAGTGGTTATCACGTTCGCCTC | 4.47E-06 |
| mature-tRNA-Val-CAC_5_end | GTTTCCGTCGTGTAGTGGTTATCACGTTCGCCTC | 0.0002473 |
| mature-tRNA-Val-CAC_5_end | GTTTCCGTGGTGTAGTGGTTATCACGTTCGCCTC | 0.001474 |
| mature-tRNA-Val-CAC_5_end | GATTCCGTAGTGTAGTGGTTATCACGTTCGCCTC | 0.0162798 |
| mature-tRNA-Val-CAC_5_end | GTTTCCGTAGTGTAGTGGATATCACGTTCGCCTC | 0.0217466 |
| mature-tRNA-Val-CAC_5_end | GTTTCCGTAGTGTAGTGGTTATCACGTTCGCGTC | 0.0048727 |
| mature-tRNA-Val-CAC_5_end | GTTTCCGTACTGTAGTGGTTATCACGTTCGCCTC | 0.0456404 |
| mature-tRNA-Val-CAC_5_end | GTTTCCGTAGTGTAGTGCTTATCACGTTCGCCTC | 2.32E-06 |
| mature-tRNA-Val-CAC_5_end | GTTTCCGTAGTGTAGTGTTTATCACGTTCGCCTC | 0.0005989 |
| mature-tRNA-Val-CAC_5_end | GTTTCCGTAGTGTAGTGATTATCACGTTCGCCTC | 2.40E-05 |
| mature-tRNA-Val-CAC_5_end | GTTTCCGTAGTGTAGTGGTTATCACGTTCGCTTC | 4.78E-05 |
| mature-tRNA-Val-CAC_5_end | GTTTCCGTAGTGTAGTGGTTATCACGTTCGCATC | 1.46E-06 |
| mature-tRNA-Val-CAC_5_end | GTTTCCGTAGTGTAGTGGTTATCACGTTCTCCTC | 0.0494052 |
| mature-tRNA-Val-CAC_5_end | GTTTCCGTAGTGTAGTGGTTATCACGTTCCCCTC | 5.77E-15 |
| mature-tRNA-Val-CAC_5_end | GTTTCCGCAGTGTAGTGGTTATCACGTTCGCCTC | 8.10E-09 |
| mature-tRNA-Val-CAC_5_end | GTTTCCGAAGTGTAGTGGTTATCACGTTCGCCTC | 2.62E-14 |
| mature-tRNA-Val-CAC_5_end | GTTTGCGTAGTGTAGTGGTTATCACGTTCGCCTC | 0.012736 |
| mature-tRNA-Val-CAC_5_end | GTTTCCGTAGTGCAGTGGTTATCACGTTCGCCTC | 9.93E-06 |
| mature-tRNA-Val-CAC_5_end | GTTTCCGGAGTGTAGTGGTTATCACGTTCGCCTC | 5.22E-12 |
| mature-tRNA-Val-CAC_5_end | GTTTCCGTAGTGAAGTGGTTATCACGTTCGCCTC | 2.22E-11 |
| mature-tRNA-Val-CAC_5_end | GTTTCCGTAGTGGAGTGGTTATCACGTTCGCCTC | 9.95E-09 |
| mature-tRNA-Val-CAC_5_end;mature-tRNA-Val-AAC_5_end | GTTTCCGTAGTTTAGTGGTTATCACGTTC | 0 |
| mature-tRNA-Val-CAC_5_end;mature-tRNA-Val-AAC_5_end | GTTTCCGTAGTATAGTGGTTATCACGTTC | 0 |
| mature-tRNA-Val-CAC_5_end;mature-tRNA-Val-AAC_5_end | GTTTCCGTAGTATAGTGGTTATCACGTTCGCC | 0 |
| mature-tRNA-Val-CAC_5_end;mature-tRNA-Val-AAC_5_end | GTTTCCGTAGTATAGTGGTTATCACGTTCG | 0 |
| mature-tRNA-Val-CAC_5_end;mature-tRNA-Val-AAC_5_end | GTTTCCGTAGTATAGTGGTTATCACGTTCGCCT | 0 |
| mature-tRNA-Val-CAC_5_end;mature-tRNA-Val-AAC_5_end | GTTTCCGTAGTCTAGTGGTTATCACGTTCG | 0 |
| mature-tRNA-Val-CAC_5_end;mature-tRNA-Val-AAC_5_end | GTTTCCGTAGTCTAGTGGTTATCACGTTC | 0 |
| mature-tRNA-Val-CAC_5_end;mature-tRNA-Val-AAC_5_end | GTTTCCGTAGTTTAGTGGTTATCACGTTCGCCT | 0 |
| mature-tRNA-Val-CAC_5_end;mature-tRNA-Val-AAC_5_end | GTTTCCGTAGTTTAGTGGTTATCACGTTCGCC | 0 |
| mature-tRNA-Val-CAC_5_end;mature-tRNA-Val-AAC_5_end | GTTTCCGTAGTCTAGTGGTTATCACGTTCGC | 0 |
| mature-tRNA-Val-CAC_5_end;mature-tRNA-Val-AAC_5_end | GTTTCCGTAGTCTAGTGGTTATCACGTTCGCC | 0 |
| mature-tRNA-Val-CAC_5_end;mature-tRNA-Val-AAC_5_end | GTTTCCGTCGTGTAGTGGTTATCACGTTCG | 0 |
| mature-tRNA-Val-CAC_5_end;mature-tRNA-Val-AAC_5_end | GTTTCCGTCGTGTAGTGGTTATCACGTTC | 0 |
| mature-tRNA-Val-CAC_5_end;mature-tRNA-Val-AAC_5_end | GTTTCCGTAGTGTAGTGGTTATGACGTTCGCCT | 5.10E-09 |
| mature-tRNA-Val-CAC_5_end;mature-tRNA-Val-AAC_5_end | GTTTCCGTAGTGTAGTGGTTATGACGTTCGCC | 5.08E-12 |
| mature-tRNA-Val-CAC_5_end;mature-tRNA-Val-AAC_5_end | GTTTCCGTCGTGTAGTGGTTATCACGTTCGCCT | 0 |
| mature-tRNA-Val-CAC_5_end;mature-tRNA-Val-AAC_5_end | GTTTCCGTAGTGTAGTGGTTATGACGTTCG | 0 |
| mature-tRNA-Val-CAC_5_end;mature-tRNA-Val-AAC_5_end | GTTTCCGTAGTGTAGTGGTTATGACGTTCGC | 0 |
| mature-tRNA-Val-CAC_5_end;mature-tRNA-Val-AAC_5_end | GTTTCCGTGGTGTAGTGGTTATCACGTTCG | 0 |
| mature-tRNA-Val-CAC_5_end;mature-tRNA-Val-AAC_5_end | GTTTCCGTGGTGTAGTGGTTATCACGTTC | 0 |
| mature-tRNA-Val-CAC_5_end;mature-tRNA-Val-AAC_5_end | GTTTCCGTGGTGTAGTGGTTATCACGTTCGC | 0 |
| mature-tRNA-Val-CAC_5_end;mature-tRNA-Val-AAC_5_end | GTTTCCGTAGTGTAGTGGTTATCTCGTTC | 0 |
| mature-tRNA-Val-CAC_5_end;mature-tRNA-Val-AAC_5_end | GTTTCCGTAGTGTAGTGGTTATCTCGTTCG | 0 |
| mature-tRNA-Val-CAC_5_end;mature-tRNA-Val-AAC_5_end | GTTTCCGTGGTGTAGTGGTTATCACGTTCGCCT | 0 |
| mature-tRNA-Val-CAC_5_end;mature-tRNA-Val-AAC_5_end | GTTGCCGTAGTGTAGTGGTTATCACGTTC | 6.39E-10 |
| mature-tRNA-Val-CAC_5_end;mature-tRNA-Val-AAC_5_end | GATTCCGTAGTGTAGTGGTTATCACGTTCG | 0 |
| mature-tRNA-Val-CAC_5_end;mature-tRNA-Val-AAC_5_end | GATTCCGTAGTGTAGTGGTTATCACGTTC | 0 |
| mature-tRNA-Val-CAC_5_end;mature-tRNA-Val-AAC_5_end | GTTTCCGTAGTGTAGTGGTTATCTCGTTCGC | 0 |
| mature-tRNA-Val-CAC_5_end;mature-tRNA-Val-AAC_5_end | GTTTCCGTAGTGTAGTGGTTTTCACGTTCG | 0.0002425 |
| mature-tRNA-Val-CAC_5_end;mature-tRNA-Val-AAC_5_end | GTTACCGTAGTGTAGTGGTTATCACGTTCGC | 4.13E-12 |
| mature-tRNA-Val-CAC_5_end;mature-tRNA-Val-AAC_5_end | GATTCCGTAGTGTAGTGGTTATCACGTTCGCCT | 1.29E-10 |
| mature-tRNA-Val-CAC_5_end;mature-tRNA-Val-AAC_5_end | GTTTCCGTAGTGTAGTGGCTATCACGTTC | 0 |
| mature-tRNA-Val-CAC_5_end;mature-tRNA-Val-AAC_5_end | GTTTCCGTAGTGTAGTGGTTATAACGTTC | 8.89E-07 |
| mature-tRNA-Val-CAC_5_end;mature-tRNA-Val-AAC_5_end | GTTTCCGTAGTGTAGTGGCTATCACGTTCG | 0 |
| mature-tRNA-Val-CAC_5_end;mature-tRNA-Val-AAC_5_end | GTTACCGTAGTGTAGTGGTTATCACGTTCGCC | 2.42E-06 |
| mature-tRNA-Val-CAC_5_end;mature-tRNA-Val-AAC_5_end | GTTGCCGTAGTGTAGTGGTTATCACGTTCGCCT | 0.0006099 |
| mature-tRNA-Val-CAC_5_end;mature-tRNA-Val-AAC_5_end | GTTTCCGTAGTGTAGTGGATATCACGTTCG | 0 |
| mature-tRNA-Val-CAC_5_end;mature-tRNA-Val-AAC_5_end | GTTACCGTAGTGTAGTGGTTATCACGTTCGCCT | 4.94E-05 |
| mature-tRNA-Val-CAC_5_end;mature-tRNA-Val-AAC_5_end | GTTTCCGTAGTGTAGTGGATATCACGTTC | 0 |
| mature-tRNA-Val-CAC_5_end;mature-tRNA-Val-AAC_5_end | GTTTCCGTAGTGTAGTGGGTATCACGTTCG | 0 |
| mature-tRNA-Val-CAC_5_end;mature-tRNA-Val-AAC_5_end | GTTTCCGTAGTGTAGTGGTTTTCACGTTCGC | 0.0353343 |
| mature-tRNA-Val-CAC_5_end;mature-tRNA-Val-AAC_5_end | GTTTCCGTAGTGTAGTGGTTATCTCGTTCGCCT | 2.13E-07 |
| mature-tRNA-Val-CAC_5_end;mature-tRNA-Val-AAC_5_end | GTTTCCGTAGTGTAGTGGATATCACGTTCGCC | 1.11E-16 |
| mature-tRNA-Val-CAC_5_end;mature-tRNA-Val-AAC_5_end | GTTTCCGTAGTGTAGTGGCTATCACGTTCGC | 0 |
| mature-tRNA-Val-CAC_5_end;mature-tRNA-Val-AAC_5_end | GTTTCCGTAGTGTAGTGGCTATCACGTTCGCC | 8.65E-09 |
| mature-tRNA-Val-CAC_5_end;mature-tRNA-Val-AAC_5_end | GTGTCCGTAGTGTAGTGGTTATCACGTTC | 0.0002843 |
| mature-tRNA-Val-CAC_5_end;mature-tRNA-Val-AAC_5_end | GTTTCCGTAGTGTAGTGGCTATCACGTTCGCCT | 4.66E-07 |
| mature-tRNA-Val-CAC_5_end;mature-tRNA-Val-AAC_5_end | GTTTCCGTAGTGTAGTGGGTATCACGTTCGCC | 1.52E-07 |
| mature-tRNA-Val-CAC_5_end;mature-tRNA-Val-AAC_5_end | GTTTCCGTAGTGTAGTGGGTATCACGTTCGC | 7.94E-14 |
| mature-tRNA-Val-CAC_5_end;mature-tRNA-Val-AAC_5_end | GTTTCCGTAGTGTAGTGGATATCACGTTCGCCT | 5.88E-10 |
| mature-tRNA-Val-CAC_5_end;mature-tRNA-Val-AAC_5_end | GTTTCCGTAGTGTAGTGGTTATAACGTTCGC | 0.0033079 |
| mature-tRNA-Val-CAC_5_end;mature-tRNA-Val-AAC_5_end | TTTTCCGTAGTGTAGTGGTTATCACGTTC | 0.0002571 |
| mature-tRNA-Val-CAC_5_end;mature-tRNA-Val-AAC_5_end | GTTTTCGTAGTGTAGTGGTTATCACGTTCGC | 1.64E-06 |
| mature-tRNA-Val-CAC_5_end;mature-tRNA-Val-AAC_5_end | GTTTCCGTAGTGTAGTGATTATCACGTTC | 0 |
| mature-tRNA-Val-CAC_5_end;mature-tRNA-Val-AAC_5_end | GTTTCCGTAGTGTAATGGTTATCACGTTCGCCT | 8.79E-08 |
| mature-tRNA-Val-CAC_5_end;mature-tRNA-Val-AAC_5_end | GTTTCCGTAGTGTAATGGTTATCACGTTCGC | 2.72E-14 |
| mature-tRNA-Val-CAC_5_end;mature-tRNA-Val-AAC_5_end | GTTTTCGTAGTGTAGTGGTTATCACGTTCGCCT | 0.001323 |
| mature-tRNA-Val-CAC_5_end;mature-tRNA-Val-AAC_5_end | GTTTTCGTAGTGTAGTGGTTATCACGTTCGCC | 4.10E-05 |
| mature-tRNA-Val-CAC_5_end;mature-tRNA-Val-AAC_5_end | GTTTCCGTAGTGTAGTGGTTATCACGTTCGCT | 0 |
| mature-tRNA-Val-CAC_5_end;mature-tRNA-Val-AAC_5_end | GTTTCCGTAGTGTAGTGGTTATCAGGTTC | 0.0259311 |
| mature-tRNA-Val-CAC_5_end;mature-tRNA-Val-AAC_5_end | GTTTCCGTAGTGTAGTGATTATCACGTTCG | 0 |
| mature-tRNA-Val-CAC_5_end;mature-tRNA-Val-AAC_5_end | GTTTCCGTACTGTAGTGGTTATCACGTTCG | 0 |
| mature-tRNA-Val-CAC_5_end;mature-tRNA-Val-AAC_5_end | GTTTCCGTAGTGTAGTGTTTATCACGTTCGCC | 0 |
| mature-tRNA-Val-CAC_5_end;mature-tRNA-Val-AAC_5_end | GTTTCCGTATTGTAGTGGTTATCACGTTCGC | 0.0009294 |
| mature-tRNA-Val-CAC_5_end;mature-tRNA-Val-AAC_5_end | GTTTCCGTATTGTAGTGGTTATCACGTTCGCCT | 0.0105406 |
| mature-tRNA-Val-CAC_5_end;mature-tRNA-Val-AAC_5_end | GTTTCCGTAGTGTAGTGGTTATCACGTTCGCA | 0 |
| mature-tRNA-Val-CAC_5_end;mature-tRNA-Val-AAC_5_end | GTTTCCGTAGTGTAGTGCTTATCACGTTCGCC | 0 |
| mature-tRNA-Val-CAC_5_end;mature-tRNA-Val-AAC_5_end | GTTTCCGTAGTGTAGTCGTTATCACGTTCGC | 0.0013937 |
| mature-tRNA-Val-CAC_5_end;mature-tRNA-Val-AAC_5_end | GTTTACGTAGTGTAGTGGTTATCACGTTCGC | 2.18E-11 |
| mature-tRNA-Val-CAC_5_end;mature-tRNA-Val-AAC_5_end | GTTTCCGTACTGTAGTGGTTATCACGTTCGC | 0 |
| mature-tRNA-Val-CAC_5_end;mature-tRNA-Val-AAC_5_end | GTTTCCGTAGTGTAGTGGTTATCACGTTCTCC | 2.81E-07 |
| mature-tRNA-Val-CAC_5_end;mature-tRNA-Val-AAC_5_end | GTTTCCGTAGTGTAGTCGTTATCACGTTCGCC | 0.0076325 |
| mature-tRNA-Val-CAC_5_end;mature-tRNA-Val-AAC_5_end | GTTTCCGTAGTGTAGTGATTATCACGTTCGC | 0 |
| mature-tRNA-Val-CAC_5_end;mature-tRNA-Val-AAC_5_end | GTTTCCGTAGTGTAGTGGTTATCACGTTCGCG | 0 |
| mature-tRNA-Val-CAC_5_end;mature-tRNA-Val-AAC_5_end | GTTTCCGTACTGTAGTGGTTATCACGTTCGCC | 0 |
| mature-tRNA-Val-CAC_5_end;mature-tRNA-Val-AAC_5_end | GTTTACGTAGTGTAGTGGTTATCACGTTCGCC | 6.49E-08 |
| mature-tRNA-Val-CAC_5_end;mature-tRNA-Val-AAC_5_end | GTTTCCGTAGTGTAGTGGTTAACACGTTC | 4.06E-08 |
| mature-tRNA-Val-CAC_5_end;mature-tRNA-Val-AAC_5_end | GTTTCCGTAGTGTAGTGATTATCACGTTCGCCT | 0 |
| mature-tRNA-Val-CAC_5_end;mature-tRNA-Val-AAC_5_end | GTTTCCGTAGTGTAGTGGTTATCACGTTCC | 0 |
| mature-tRNA-Val-CAC_5_end;mature-tRNA-Val-AAC_5_end | GTTTCCGTAGTGTAGTGGTTATCACGTTCTCCT | 6.25E-12 |
| mature-tRNA-Val-CAC_5_end;mature-tRNA-Val-AAC_5_end | GTTTCCGTAGTGTAGTGGTTATCAGGTTCGC | 0.0001429 |
| mature-tRNA-Val-CAC_5_end;mature-tRNA-Val-AAC_5_end | GTTTCCGTAGTGTAGTGGTTATCACGTTCTC | 0 |
| mature-tRNA-Val-CAC_5_end;mature-tRNA-Val-AAC_5_end | GTTTCCGCAGTGTAGTGGTTATCACGTTC | 0 |
| mature-tRNA-Val-CAC_5_end;mature-tRNA-Val-AAC_5_end | GTTTCCGTAGTGTAGTGGTCATCACGCTCCCCT | 0.0032239 |
| mature-tRNA-Val-CAC_5_end;mature-tRNA-Val-AAC_5_end | GTTTCCGCAGTGTAGTGGTTATCACGTTCG | 0 |
| mature-tRNA-Val-CAC_5_end;mature-tRNA-Val-AAC_5_end | GTTTCCGCAGTGTAGTGGTTATCACGTTCGCC | 0 |
| mature-tRNA-Val-CAC_5_end;mature-tRNA-Val-AAC_5_end | GTTTCCGTAGTGTAGTGGTTAACACGTTCGCC | 1.59E-07 |
| mature-tRNA-Val-CAC_5_end;mature-tRNA-Val-AAC_5_end | GTTTCCGTAGTGTAGTGGTCATCACGCTCCC | 0.0087685 |
| mature-tRNA-Val-CAC_5_end;mature-tRNA-Val-AAC_5_end | GTTTCCGTAGTGTAGTGGTTATCACGTTCCCC | 0 |
| mature-tRNA-Val-CAC_5_end;mature-tRNA-Val-AAC_5_end | GTTTCCGTAGTGTAGTGGTTATCACGTTCGCTT | 0 |
| mature-tRNA-Val-CAC_5_end;mature-tRNA-Val-AAC_5_end | GTTTCCGCAGTGTAGTGGTTATCACGTTCGC | 0 |
| mature-tRNA-Val-CAC_5_end;mature-tRNA-Val-AAC_5_end | GTTTCCGTAGTGTAGTGGTTATCACGTTCCCCT | 0 |
| mature-tRNA-Val-CAC_5_end;mature-tRNA-Val-AAC_5_end | GTTTCCGTAGTGTAGTGGTTAACACGTTCGCCT | 2.29E-09 |
| mature-tRNA-Val-CAC_5_end;mature-tRNA-Val-AAC_5_end | GTTTCCGAAGTGTAGTGGTTATCACGTTCGC | 0 |
| mature-tRNA-Val-CAC_5_end;mature-tRNA-Val-AAC_5_end | GTTTCCGTAGTGCAGTGGTTATCACGTTC | 0 |
| mature-tRNA-Val-CAC_5_end;mature-tRNA-Val-AAC_5_end | GTTTCCGTAGTGTAGTGGTTAACACGTTCGC | 0 |
| mature-tRNA-Val-CAC_5_end;mature-tRNA-Val-AAC_5_end | GTTTCCGGAGTGTAGTGGTTATCACGTTC | 0 |
| mature-tRNA-Val-CAC_5_end;mature-tRNA-Val-AAC_5_end | GTTTCCGTAGTGTAGTGGTTATCACGTTCCC | 0 |
| mature-tRNA-Val-CAC_5_end;mature-tRNA-Val-AAC_5_end | GTTTCCGGAGTGTAGTGGTTATCACGTTCG | 0 |
| mature-tRNA-Val-CAC_5_end;mature-tRNA-Val-AAC_5_end | GTTTCCGTAGTGCAGTGGTTATCACGTTCGCC | 0 |
| mature-tRNA-Val-CAC_5_end;mature-tRNA-Val-AAC_5_end | GTTTCCGGAGTGTAGTGGTCATCACGCTCGCCT | 0.0463738 |
| mature-tRNA-Val-CAC_5_end;mature-tRNA-Val-AAC_5_end | GTTTCCGAAGTGTAGTGGTCATCACGCTCGCC | 0.0243621 |
| mature-tRNA-Val-CAC_5_end;mature-tRNA-Val-AAC_5_end | GTTTCCGGAGTGTAGTGGTTATCACGTTCGC | 0 |
| mature-tRNA-Val-CAC_5_end;mature-tRNA-Val-AAC_5_end | GTTTCCGTAGTGCAGTGGTTATCACGTTCGC | 0 |
| mature-tRNA-Val-CAC_5_end;mature-tRNA-Val-AAC_5_end | GTTTCCGGAGTGTAGTGGTTATCACGTTCGCCT | 0 |
| mature-tRNA-Val-CAC_5_end;mature-tRNA-Val-AAC_5_end | GTTTCCGTAGTGCAGTGGTTATCACGTTCGCCT | 0 |
| mature-tRNA-Val-CAC_5_end;mature-tRNA-Val-AAC_5_end | GTTTCCGTAGTGAAGTGGTTATCACGTTC | 0 |
| mature-tRNA-Val-CAC_5_end;mature-tRNA-Val-AAC_5_end | GTTTGCGTAGTGTAGTGGTTATCACGTTCGCCT | 0 |
| mature-tRNA-Val-CAC_5_end;mature-tRNA-Val-AAC_5_end | GTTTCCGTAGTGAAGTGGTTATCACGTTCG | 0 |
| mature-tRNA-Val-CAC_5_end;mature-tRNA-Val-AAC_5_end | GTTTCCGTAGTGAAGTGGTTATCACGTTCGCC | 0 |
| mature-tRNA-Val-CAC_5_end;mature-tRNA-Val-AAC_5_end | GTTTCCGTAGTGAAGTGGTTATCACGTTCGC | 0 |
| mature-tRNA-Val-CAC_5_end;mature-tRNA-Val-AAC_5_end | GTTTCCGTAGTGGAGTGGTTATCACGTTC | 0 |
| mature-tRNA-Val-CAC_5_end;mature-tRNA-Val-AAC_5_end | GTTTCCGTAGTGGAGTGGTTATCACGTTCG | 0 |
| mature-tRNA-Val-CAC_5_end;mature-tRNA-Val-AAC_5_end | GTTTCCGTAGTGAAGTGGTTATCACGTTCGCCT | 0 |
| mature-tRNA-Val-CAC_5_end;mature-tRNA-Val-AAC_5_end | GTTTCCGTAGTGGAGTGGTTATCACGTTCGCC | 0 |
| mature-tRNA-Val-CAC_5_end;mature-tRNA-Val-AAC_5_end | GTTTCCGTAGTGGAGTGGTTATCACGTTCGC | 0 |
| mature-tRNA-Val-TAC_5_end | GGTTCCATAGTATAGCGGTTATCACGTCTGCTTT | 0 |
| mature-tRNA-Val-TAC_5_end | GGTTCCATAGTTTAGCGGTTATCACGTCTGCTTT | 0 |
| mature-tRNA-Val-TAC_5_end | GGTTCCATAGTATAGCGGTTATCACGTCTGCT | 0.0466795 |
| mature-tRNA-Val-TAC_5_end | GGTTCCATAGTCTAGCGGTTATCACGTCTGCTTT | 0 |
| mature-tRNA-Val-TAC_5_end | GGTTCCATAGTGTAGCGGTTATGACGTCTGCTTT | 1.11E-16 |
| mature-tRNA-Val-TAC_5_end | GGTTCCATCGTGTAGCGGTTATCACGTCTGCTTT | 0 |
| mature-tRNA-Val-TAC_5_end | GGTTCCATGGTGTAGCGGTTATCACGTCTGCTTT | 0 |
| mature-tRNA-Val-TAC_5_end | GGTTCCTTAGTGTAGCGGTTATCACGTCTGCTTT | 1.09E-07 |
| mature-tRNA-Val-TAC_5_end | GGTTCCATAGTGTAGCGGTTATCTCGTCTGCTTT | 0 |
| mature-tRNA-Val-TAC_5_end | GGTTCCCTAGTGTAGCGGTTATCACGTCTGCTTT | 0.0282879 |
| mature-tRNA-Val-TAC_5_end | GGTACCATAGTGTAGCGGTTATCACGTCTGCTTT | 8.95E-09 |
| mature-tRNA-Val-TAC_5_end | GGTGCCATAGTGTAGCGGTTATCACGTCTGCTTT | 2.52E-05 |
| mature-tRNA-Val-TAC_5_end | GGTTCCATAGTGTAGCGGGTATCACGTCTGCT | 0.0151061 |
| mature-tRNA-Val-TAC_5_end | GATTCCATAGTGTAGCGGTTATCACGTCTGCTTT | 0 |
| mature-tRNA-Val-TAC_5_end | GGTTCCATAGTGTAGCGGCTATCACGTCTGCTT | 0.0377853 |
| mature-tRNA-Val-TAC_5_end | GGTTCCATAGTGTAGCGGATATCACGTCTGCTT | 0.0011733 |
| mature-tRNA-Val-TAC_5_end | GGTTCCATAGTGTAGCGGATATCACGTCTGCTTT | 0 |
| mature-tRNA-Val-TAC_5_end | GGTTCCATAGTGTAGCGGTTATAACGTCTGCTTT | 0.0097509 |
| mature-tRNA-Val-TAC_5_end | GGTTCCATAGTGTAGCGGATATCACGTCTGCT | 0.0015123 |
| mature-tRNA-Val-TAC_5_end | GGTTCCATAGTGTAGCGGGTATCACGTCTGCTTT | 0 |
| mature-tRNA-Val-TAC_5_end | GGTTCCATAGTGTAGCGGCTATCACGTCTGCT | 0.0437984 |
| mature-tRNA-Val-TAC_5_end | GGTTCCATAGTGTAGCGGCTATCACGTCTGCTTT | 0 |
| mature-tRNA-Val-TAC_5_end | GGTTCCATAGTGTTGCGGTTATCACGTCTGCTTT | 0.0018177 |
| mature-tRNA-Val-TAC_5_end | GGTTCCATAGTGTAGTGGTTATCACGTCTGCTTT | 4.30E-10 |
| mature-tRNA-Val-TAC_5_end | GGTTCCATAGTGTAGCGGTTATCACGTCTGGTTT | 6.06E-05 |
| mature-tRNA-Val-TAC_5_end | GGTTTCATAGTGTAGCGGTTATCACGTCTGCTTT | 7.26E-05 |
| mature-tRNA-Val-TAC_5_end | GGTTCCATAGTGTAGAGGTTATCACGTCTGCTTT | 0.0047046 |
| mature-tRNA-Val-TAC_5_end | GGTTCCATAGTGTAGCGTTTATCACGTCTGCTTT | 0 |
| mature-tRNA-Val-TAC_5_end | GGTTCCATAGTGTAGCGGTTATCACGTCTACTTT | 7.33E-15 |
| mature-tRNA-Val-TAC_5_end | GGTTCCATAGTGTAGCGGTTATCAGGTCTGCTTT | 0.0042716 |
| mature-tRNA-Val-TAC_5_end | GGTTCCATAGTGTAGCGCTTATCACGTCTGCTTT | 0 |
| mature-tRNA-Val-TAC_5_end | GGTTCCATAGTGTAGCGCTTATCACGTCTGCT | 0.0029431 |
| mature-tRNA-Val-TAC_5_end | GGTTCCATACTGTAGCGGTTATCACGTCTGCTTT | 0 |
| mature-tRNA-Val-TAC_5_end | GGTTCCATAGTGTAGCGGTTATCACGTCTTCTTT | 3.11E-10 |
| mature-tRNA-Val-TAC_5_end | GGTTACATAGTGTAGCGGTTATCACGTCTGCTTT | 2.59E-11 |
| mature-tRNA-Val-TAC_5_end | GGTTCCATAGTGTAGCGCTTATCACGTCTGCTT | 0.0084785 |
| mature-tRNA-Val-TAC_5_end | GGTTCCATAGTGTAGCGCTTATCACGTCTGC | 0.0151844 |
| mature-tRNA-Val-TAC_5_end | GGTTCCATAGTGTAGCGCTTATCACGTCTG | 0.0459272 |
| mature-tRNA-Val-TAC_5_end | GGTTCCATAGTGTAGCGATTATCACGTCTGCTTT | 0 |
| mature-tRNA-Val-TAC_5_end | GGTTCCATAGTGTAGCGCTTATCACGTCT | 0.0157469 |
| mature-tRNA-Val-TAC_5_end | GGTTCCATAGTGTAGCGTTTATCACGTCTGCTT | 0.0494052 |
| mature-tRNA-Val-TAC_5_end | GGTTCCATAGTGTAGCGGTTAACACGTCTGCTTT | 4.45E-08 |
| mature-tRNA-Val-TAC_5_end | GGTTCCATAGTGTAGCGGTTATCACGTCTGCGTT | 0.0002101 |
| mature-tRNA-Val-TAC_5_end | GGTTCCATAGTGTAGCGGTTATCACGTCTGCATT | 4.50E-05 |
| mature-tRNA-Val-TAC_5_end | GGTTCCATAGTGTAGCGATTATCACGTCTGCT | 0.0087685 |
| mature-tRNA-Val-TAC_5_end | GGTTCCATAGTGTAGCGGTTAGCACGTCTGCTTT | 0.0054155 |
| mature-tRNA-Val-TAC_5_end | GGTTCCATAGTGTAGCGATTATCACGTCTGCTT | 0.0494052 |
| mature-tRNA-Val-TAC_5_end | GGTTCCATAGTGCAGCGGTTATCACGTCTGCTTT | 0 |
| mature-tRNA-Val-TAC_5_end | GGTTCCATAGTGTAGCGGTTATCACGTCTCCT | 0.0243621 |
| mature-tRNA-Val-TAC_5_end | GGTTCCATAGTGTAGCGGTTATCACGTCTCCTT | 0.0243621 |
| mature-tRNA-Val-TAC_5_end | GGTTCCATAGTGTAGCGGTTATCACGTCTCCTTT | 0 |
| mature-tRNA-Val-TAC_5_end | GGTTCCAAAGTGTAGCGGTTATCACGTCTGCTTT | 0 |
| mature-tRNA-Val-TAC_5_end | GGTTCCACAGTGTAGCGGTTATCACGTCTGCTTT | 0 |
| mature-tRNA-Val-TAC_5_end | GGTTGCATAGTGTAGCGGTTATCACGTCTGCTTT | 0 |
| mature-tRNA-Val-TAC_5_end | GGTTCCATAGTGAAGCGGTTATCACGTCTGCT | 0.0066316 |
| mature-tRNA-Val-TAC_5_end | GGTTCCATAGTGAAGCGGTTATCACGTCTGCTT | 0.0066316 |
| mature-tRNA-Val-TAC_5_end | GGTTCCATAGTGAAGCGGTTATCACGTCTGCTTT | 0 |
| mature-tRNA-Val-TAC_5_end | GGTTCCATAGTGGAGCGGTTATCACGTCTGCTTT | 0 |
| mature-tRNA-Val-TAC_5_end | GGTTCCATAGTGAAGCGGTTATCACGTCTGC | 0.0243621 |
| mature-tRNA-Val-TAC_5_end | GGTTCCAGAGTGTAGCGGTTATCACGTCTGCTTT | 0 |

**Supplementary Table 4** Summary of outcome after sperm 30-40 nt RNAs and water injection into normal zygotes.

|  | Sperm 29-34nt RNAs injection | | | Control injection（TE） | | |  |
| --- | --- | --- | --- | --- | --- | --- | --- |
|  | Injected zygotes | Transferred zygotes | Live born (% transfer) | Injected zygotes | Transferred zygotes | Live born (% transfer) |  |
| Con | 75 | 75 | 34(45%) | 75 | 75 | 30 (40%) |  |
| H2O2 | 75 | 75 | 35 (47%) |  |  |  |  |
| Note: For embryo transfer, zygotes (10-15) were transferred into one side of oviduct, both oviducts get transfers, with an amount of 25 zygotes transferred each surrogate mother. Four mice were used as surrogate mother every groups. | | | | | | |  |
|  |  |  |  |  |  |  |  |
|  |  |  |  |  |  |  |  |

**Supplementary Table 5** Body composition and fasting blood glucose in F1 male mice at 17 weeks of age

|  | Con-F1 | OS-F1 | Con-F1 (% body weight) | OS-F1 (% body weight) |
| --- | --- | --- | --- | --- |
| Body weight (g) | 26.723±0.292 | 26.403±0.313 |  |  |
| Brown fat (g) | 0.098±0.005 | 0.108±0.005 | 0.365±0.018 | 0.408±0.016 |
| Subcutaneous fat (g) | 0.214±0.016 | 0.203±0.012 | 0.799±0.059 | 0.770±0.045 |
| Epididymis fat (g) | 0.482±0.034 | 0.544±0.037 | 1.807±0.125 | 2.042±0.120 |
| Gastrocnemius (g) | 0.295±0.003 | 0.298±0.005 | 1.103±0.010 | 1.129±0.010 |
| Liver (g) | 1.034±0.022 | 1.052±0.018 | 3.875±0.093 | 3.984±0.075 |
| Kidney (g) | 0.317±0.008 | 0.315±0.005 | 1.184±0.024 | 1.194±0.020 |
| Testes (g) | 0.179±0.003 | 0.174±0.004 | 0.669±0.011 | 0.661±0.018 |
| Glucose (mM) | 6.711±0.230 | 7.777±0.331* |  |  |

**Supplementary Table 6** Body composition and fasting blood glucose in F1 female mice at 17 weeks of age

|  | Con-F1 | OS-F1 | Con-F1 (% body weight) | OS-F1 (% body weight) |
| --- | --- | --- | --- | --- |
| Body weight (g) | 20.286±0.288 | 19.871±0.292 |  |  |
| Brown fat(g) | 0.046±0.002 | 0.049±0.002 | 0.225±0.011 | 0.248±0.011 |
| Subcutaneous fat(g) | 0.162±0.008 | 0.122±0.008** | 0.799±0.039 | 0.614±0.043** |
| Perivitelline fat(g) | 0.238±0.017 | 0.213±0.015 | 1.167±0.076 | 1.068±0.068 |
| Gastrocnemius(g) | 0.211±0.004 | 0.215±0.004 | 1.042±0.014 | 1.081±0.014 |
| Liver(g) | 0.888±0.018 | 0.893±0.026 | 4.380±0.066 | 4.497±0.114 |
| Kidney(g) | 0.262±0.004 | 0.271±0.006 | 1.216±0.077 | 1.363±0.021 |
| Spleen(g) | 0.059±0.002 | 0.072±0.004* | 0.275±0.02 | 0.363±0.021** |
| Glucose (mM) | 3.832±0.166 | 3.411±0.161 |  |  |
